# Hollow-fiber bioreactor production of extracellular vesicles from human bone marrow mesenchymal stromal cells yields nanovesicles that mirrors the immuno-modulatory antigenic signature of the producer cell

**DOI:** 10.1186/s13287-021-02190-3

**Published:** 2021-02-12

**Authors:** Jonathan Gobin, Gauri Muradia, Jelica Mehic, Carole Westwood, Lauren Couvrette, Andrew Stalker, Stewart Bigelow, Christian C. Luebbert, Frédéric St-Denis Bissonnette, Michael J. W. Johnston, Simon Sauvé, Roger Y. Tam, Lisheng Wang, Michael Rosu-Myles, Jessie R. Lavoie

**Affiliations:** 1 Department of Biochemistry, Microbiology, and Immunology, University of Ottawa, Ottawa, Ontario Canada; 2 Centre for Biologics Evaluation, Biologic and Radiopharmaceutical Drugs Directorate, Health Products and Food Branch, Health Canada, Ottawa, Ontario Canada; 3 Department of Chemistry, Carleton University, Ottawa, Ontario Canada

**Keywords:** Human mesenchymal stromal cells, Extracellular vesicles, Hollow-fiber bioreactor system, Immune-profiling, Glycan, cGMP-compliant environment

## Abstract

**Background:**

Extracellular vesicles (EVs) produced by human bone marrow-derived mesenchymal stromal cells (hBM-MSCs) are currently investigated for their clinical effectiveness towards immune-mediated diseases. The large amounts of stem cell-derived EVs required for clinical testing suggest that bioreactor production systems may be a more amenable alternative than conventional EV production methods for manufacturing products for therapeutic use in humans.

**Methods:**

To characterize the potential utility of these systems, EVs from four hBM-MSC donors were produced independently using a hollow-fiber bioreactor system under a cGMP-compliant procedure. EVs were harvested and characterized for size, concentration, immunophenotype, and glycan profile at three separate intervals throughout a 25-day period.

**Results:**

Bioreactor-inoculated hBM-MSCs maintained high viability and retained their trilineage mesoderm differentiation capability while still expressing MSC-associated markers upon retrieval. EVs collected from the four hBM-MSC donors showed consistency in size and concentration in addition to presenting a consistent surface glycan profile. EV surface immunophenotypic analyses revealed a consistent low immunogenicity profile in addition to the presence of immuno-regulatory CD40 antigen. EV cargo analysis for biomarkers of immune regulation showed a high abundance of immuno-regulatory and angiogenic factors VEGF-A and IL-8.

**Conclusions:**

Significantly, EVs from hBM-MSCs with immuno-regulatory constituents were generated in a large-scale system over a long production period and could be frequently harvested with the same quality and quantity, which will circumvent the challenge for clinical application.

**Supplementary Information:**

The online version contains supplementary material available at 10.1186/s13287-021-02190-3.

## Introduction

Intrinsic properties of extracellular vesicles (EVs) have rendered them attractive biocompatible therapeutic nanovesicles under their native form or engineered as gene/vaccine/drug delivery systems [1, 2]. EVs are cell-released nano-sized membrane vesicles that can distribute systematically and cross the blood-brain barrier to act as mediators of cell-cell communication [3–5]. Through the transfer of their bioactive payload (cytokines, growth factors, signaling lipids, mRNAs, and regulatory miRNAs) and/or through the binding via their membrane-bound molecules (receptors, lipids, integrins, and glycans), EVs can regulate cell/tissue responses, and more broadly, have immune-modulating effects [6–10]. Their native membrane constituents and intrinsic abilities to be transferred from one cell to another may play a role in their enhanced bioavailability and lower immunogenicity and prospects as a new biological nanoplatforms for drug delivery or diagnostic purpose [11, 12].

The clinical implementation of EV-based therapies derived from cultured cells relies on large quantities of high-quality EVs produced by viable cells maintained under well-controlled conditions. The traditional flask-based culture method to produce EVs from adherent cells does not allow for a continuous production of large quantities of the biological product and therefore precludes their use for clinical preparations. On the other hand, bioreactor systems overcome this shortfall, while providing the necessary environment to maintain high cell viability and homeostasis [13–15]. EV production based on a bioreactor system presents several benefits where scalability, reduced manual handling, and easy monitoring and control of culture parameters can be achieved [13, 16]. Furthermore, the use of a bioreactor can increase the translational value of biotherapeutics as this environment is a better representation of the cell-cell interaction found in vivo as compared to the flask-based method [17]. The hollow-fiber bioreactor from FiberCell Systems allows for seeding large amounts of adherent cells based on its hollow-fiber technology which consequently increases the cell seeding surface area (medium-sized cartridge offers 4000 cm^2^ of surface area) [18, 19]. In turn, this configuration allows for a scale-up of continuous EV production sampled over time from the EV-rich cell-conditioned medium produced by millions of cells, thus allowing anticipated clinical doses to be achieved. While EVs have previously been produced using hollow fibers, they were only done on a 48-h production scheme using human embryonic kidney (HEK293) cells [14]. To test the feasibility of scale up applications with primary human bone marrow-derived mesenchymal stromal cells (hBM-MSCs) into the hollow-fiber system, we extended the EV production to 25 days.

Human BM-MSC-EVs are now considered for clinical testing where their safety and efficacy profiles are explored for the treatment of acute ischemic stroke (Clinicaltrial.gov Identifier, NCT03384433), type I diabetes mellitus (NCT02138331), macular holes (NCT03437759), dry eye symptoms in patients with chronic graft-versus-host-disease (GvHD) (NCT04213248), and more recently, for the treatment of patients hospitalized with severe novel coronavirus pneumonia (NCT04276987). These trials are based on the premise that the EVs derived from human MSCs are the mediators of the sought therapeutic action of the cells (immune-modulatory and anti-inflammatory effects) and that cell-free therapy is safer than cell-based therapies [2, 20]. Furthermore, a published report from Kordelas et al. showed that human MSC-EVs are a promising treatment modality for graft-versus-host-disease (GvHD) [21], an indication already approved by regulatory agencies for the use of hMSCs for its management [22]. In the Kordelas report, the patient with therapy-refractory GvHD showed overall improvements on the reported clinical GvHD symptoms after being administered EVs every 2 to 3 days for a total of four dosages (each dose was the equivalent of 4 × 10^6^ EVs). Furthermore, a recently published nonrandomized open-label cohort study by Sengupta and colleagues evaluated both the safety and efficacy profile of administered MSC-EVs to twenty-four patients with SARS-CoV-2-associated acute respiratory distress syndrome [23]. Patients’ clinical status improved significantly as evidenced by restoration of oxygenation and improvements of inflammation and immunocompetence [23], making human MSC-EVs not only a promising therapeutic option for GvHD but for other inflammatory/immune diseases [21].

Establishing clinically compatible and meaningful options for the production of EVs from human MSCs will contribute towards the successful translation of safe and effective EV-based therapeutic products into the clinic. Essential requirements for the translation of EV-based therapies rely on the characteristics of the producer cells, which is in turn, are influenced by their culture environment. EVs not only retain a molecular signature from the parental cell from which they are released, but the physico-chemical properties of EVs may also be influenced by the culture environment in which they are produced [24, 25]. Therefore, monitoring the overall cell health status of the EV producer cells throughout the incubation time in a bioreactor system is a vital requirement for producing EVs that will present molecular constituents that are reflective of a normal healthy cell. Producing EVs under a well-controlled culture environment, such as a cGMP-compliant setting using xeno- and serum-free conditions, will aid in producing EVs that do not elicit unwanted immunological reactions. This is most particularly important when treating patients with immune system disorders and autoimmune diseases, indications for which human MSC-EVs are currently being tested at the clinical trial stage. Furthermore, achieving desirable EV dosages for treatment relies on a high cell-density culture of human MSCs to attain anticipated EV quantities. For example, administered doses of cord blood-derived human MSC-EVs for type I diabetes mellitus in clinical trial #NCT02138331 consists of an EV dose isolated from the equivalent of 1.2–1.5 × 10^6^ cells/kg. Thus, for a patient of 60 kg enrolled in this trial, the EV dose required would therefore be produced by 7.3–9.1 × 10^7^ cells. Another example is registered trial #NCT04276987 for the treatment of patients hospitalized with severe novel coronavirus pneumonia who will receive 5 aerosol inhalations of MSC-derived exosomes (or EVs) (2.0 × 10^8^ EVs/3 mL) on a daily basis.

Here we show for the first time a comprehensive study providing extensive characterization and immunophenotypic profiling of human MSC-EVs manufactured in the hollow-fiber system from FiberCell Systems under cGMP-compliant conditions over a 25-day period. Notably, EVs produced using this bioreactor technology showed desirable translatable clinical features where they consistently displayed a low immunogenicity and immuno-regulatory antigenic signature that mirrors the immuno-modulatory antigenic signature of the producer cell. Significantly, EVs with immuno-regulatory constituents were generated in a large-scale system over a long production period and could be frequently harvested with the same quality and quantity, which will circumvent the challenge for clinical application.

## Materials and methods

### hBM-MSC cultures

Human bone marrow mesenchymal stromal cells (hBM-MSCs) were derived from the bone marrow of four healthy male donors which were generated by the company RoosterBio (RB) Inc. (Frederick, MD, USA) under informed consent (Supplementary Table 1). The purchased hBM-MSC cultures were fully characterized according to the International Society for Cell and Gene Therapy’s (ISCT) minimal criteria [26]. Our laboratory further performed hBM-MSC characterization for the expression of surface markers by flow cytometry and the trilineage mesoderm differentiation potential (adipocytes, osteocytes, and chondrocytes) (Supplementary Table 1). The hBM-MSC cultures from the four donors were expanded from initial purchase at passages 0–1 to generate working cell banks at passages 1–2 as per RoosterBio Inc.’s protocol with slight modifications, as previously described in Cheung et al. [27]. hBM-MSCs were grown in RB complete medium composed of hBM-MSC high-performance basal medium and hBM-MSC Media Booster GTX supplement (cat.#KT-001, RoosterBio Inc.).

### Large-scale expansion of hBM-MSCs in CellSTACK culture chambers

For large-scale expansion in CellSTACK culture chambers (10-stack), 20 million hBM-MSCs from the working cell bank described in the “hBM-MSC cultures” section were seeded in the chambers at a seeding density of 3145 cells/cm^2^ (Corning, cat.#3271). In this system, a complete RoosterBio culture medium consisting of RoosterBio-MSC basal medium (RoosterBio cat.#SU-005) and RoosterBooster (RoosterBio, cat.# SU-003) was prepared as recommended by the manufacturer’s protocol (i.e., 250 mL RoosterBio basal medium was mixed with 5 mL RoosterBooster). In parallel, a T-175 flask was seeded using the same cell seeding density of 3145 cells/cm^2^ (total of 550,375 cells per flask) using a complete RoosterBio culture medium in order to monitor the cell growth and morphology as cells cannot be visualized using the CellSTACK system. Cells were grown for 4 days until cell confluency reached approximately 80–90%. Cell harvesting from the CellSTACK flasks was performed as follows: medium was removed and 200 mL of Trypsin-EDTA 0.25% (Gibco, cat.#252000-72) was added and incubated at 37 °C for 6–8 min. Two hundred milliliters of 2% MSC-screened FBS (Hyclone, cat.#SH300700.03 M) prepared in D-PBS^−/−^ (Gibco, cat.#14190250) was then added to quench the trypsin activity. The cell suspension was collected in 50-mL centrifuge tubes, then centrifuged at 200×*g* for 10 min, resuspended in a final volume of 20 mL of RB complete medium and injected into the hollow-fiber bioreactor system as described in the “Hollow-fiber bioreactor system and hBM-MSC inoculation” section. The cells from the matching T-175 flask processed in parallel were handled in a similar manner to allow approximating the number of cells in the CellSTACK culture chambers. For the T-175 flask, the volume of trypsin and quenching reagent was adjusted to 7.5 mL of each reagent and the cells were resuspended in 2 mL of complete medium for counting. Cell counts from both conditions (T-175 and CellSTACK) were conducted using Trypan Blue exclusion kit (Invitrogen, cat. #T10282).

### Hollow-fiber bioreactor system and hBM-MSC inoculation

CellSTACK expanded hBM-MSCs (prepared according to the “Large-scale expansion of hBM-MSCs in CellSTACK culture chambers” section) from each donor were seeded in separate hollow-fiber bioreactors (FiberCell Systems, cat.#P3202) at 90-220 × 10^6^ cells/cartridge (20-kD MWCO, 4000 cm^2^, polysulfone fiber cartridge; FiberCell Systems cat.#C2011) and maintained in RoosterCollect-EV xeno-free medium (RoosterBio Inc., cat.#M2001). The hollow-fiber bioreactor system was prepared and used according to the manufacturer’s procedure. All pre-inoculation steps were performed using sterile D-PBS^−/−^ (Gibco, cat.#14190250). The RoosterBio complete medium composed of Rooster Basal MSC medium (RoosterBio Inc., cat.#SU-005) mixed with RoosterBooster (RoosterBio Inc., cat.#SU-003) was prepared according to the manufacturer’s protocol. Prior to the injection of the cell suspension, 1 mL of media was drawn from the media reservoir to verify total glucose content using a glucose meter (AccuCheck Guide Glucose meter, Model 930) and L(+)-Lactate using the L-Lactate Assay Kit (Abcam, cat.# ab65331) (50 μL of media diluted 1000x was used). To inoculate the cells in the bioreactor system, the cell suspension (20 mL) prepared as described in the “Large-scale expansion of hBM-MSCs in CellSTACK culture chambers” section was injected into the cartridge following the manufacturer’s procedure. As per the manufacturer’s recommendation, the flow rate was set to 22 for the first 2–3 days of the 28-day cell inoculation period. From days 3–17 of the 28-day cell inoculation period into the bioreactor, the media volume in the extracellular capillary space is 250 mL and circulates at a system flow rate of 25. After day 17, the media volume is doubled to 500 mL with the same flow rate. A 1-mL aliquot of the medium from the media reservoir was collected every 2–3 days to monitor the glucose content and pH. An aliquot of 20 mL of the medium from the extracapillary space was harvested daily and immediately centrifuged at 200×*g* for 10 min and stored at − 80 °C for future EV processing. Pre-warmed RoosterCollect-EV medium (20 mL) was injected each time prior to the harvesting of the 20 mL of EV-rich cell-conditioned medium to replenish the volume retrieved. At the last day of EV production (day 25), PBS was pushed through instead of the medium as cells were retrieved after this last sampling. At the end of the EV production period of 25 days, the hBM-MSCs were retrieved using 40 mL of Trypsin-EDTA 0.25% in the extracapillary space and incubated for 10 min at 37 °C. The trypsinized cells were pushed through using PBS until 60 mL of cell suspension was obtained. The harvested cell suspension was quenched with an equivalent volume of 2% MSC-screened FBS prepared in D-PBS^−/−^. Cells were centrifuged at 200×*g* for 10 min and used for cell viability counts using the Trypan Blue exclusion kit, before being processed for downstream analyses.

### hBM-MSC trilineage mesoderm differentiation potential analysis

hBM-MSCs were assessed for trilineage mesoderm differentiation capacity after the incubation period in the hollow-fiber bioreactor system. For assessing hBM-MSC chondrogenic differentiation potential, StemX Vivo human chondrogenic supplement 100X (R&D Systems; cat.#CCM006) was added to the StemX Vivo chondrogenic base medium (R&D Systems; cat.#CCM005) according to the manufacturer’s procedure. Two hundred thousand hBM-MSCs were pelleted and incubated with 0.5 mL of chondrogenic differentiation medium for 14–28 days. Adipogenic differentiation potential was achieved by first seeding hBM-MSCs in 24-well plates (2.5 × 10^4^/well) in RoosterBio complete medium for 3 days, and then switching the medium to the adipogenic differentiation medium (R&D Systems; StemX Vivo Adipogenic Base cat.#CCM007 and the StemX Vivo Adipogenic Supplement 100X cat.#CCM011) for 14–21 days. Osteogenic differentiation potential was achieved by first seeding hBM-MSCs in 24-well plates (5.0 × 10^3^/well) in RoosterBio complete medium for 3 days and then switching the medium to the osteogenic differentiation medium (R&D Systems; StemX Vivo Osteogenic Base cat.#CCM007 and the StemX Vivo osteogenic Supplement 20X, cat.#CCM008) for 14–21 days. The culture medium was changed every 2–3 days for all three lineages using the corresponding inductive media and non-differentiated wells were kept as controls where a normal medium was used for the media change. Cells for all conditions were fixed with 4% paraformaldehyde in D-PBS^−/−^ and stained for the presence of adipocytes using Oil Red O stain (Electron Microscopy Sciences, cat#26503-02), osteocytes using Alizarin S 0.2% Solution (Electron Microscopy Sciences, cat.#26206-01), and chondrocytes using Alcian Blue 8GX (Sigma, cat.#A5268-10G). Transmitted light images were taken using Zeiss microscope using 10 and 20X objectives.

### Immunophenotyping analysis of hBM-MSCs by flow cytometry

The expression of hBM-MSC surface markers set by the ISCT’s minimal criteria for MSC characterization was analyzed using the BD Stemflow hMSC Analysis Kit (BD Biosciences, cat.#562,245) according to the manufacturer’s protocol (Supplementary Table 1). The hBM-MSCs were assessed for the panel of surface markers prior to inoculation in the hollow-fiber bioreactor and again after their 28-day incubation period, as previously described [6]. Collected cells were washed using D-PBS^−/−^ with 2% FBS (i.e., flow buffer), counted, and suspended in 1 mL of flow buffer followed by a filtration step through a 40-μm cell strainer to remove potential cell clumps. Hundred microliters of cell suspension was then added to each flow tube (0.5 × 10^6^ cells per tube) containing the antibody or cocktail of antibodies of interest as well as the corresponding isotype controls. Each tube was incubated in the dark for 30 min at 4 °C after which the cells were washed two times with the flow buffer and the volume was brought up to 4 mL with the flow buffer and cells were centrifuged at 1100 rpm for 6 min at 4 °C. The supernatant was discarded and the pellet was suspended in 0.5 mL of flow buffer and analyzed by flow cytometry using the LSRII flow cytometer (BD Biosciences). Fifty thousand events per sample were collected and raw data was analyzed using FlowJo V10 (FlowJo LLC, Ashland, OR, USA). Additional identical antibodies from the BD Stemflow hMSC Analysis Kit were purchased to analyze the expression of single CD34 and HLA-DR-stained cells.

### EV purification from the EV-rich hBM-MSC-conditioned medium collected from the hollow-fiber bioreactor system

To purify EVs, each 20-mL aliquot of the EV-rich cell-conditioned medium (CCM) harvested from the hollow-fiber system was processed by precipitation based on the Total Exosome Isolation Reagent, as previously described by our group [6], for the exception of the CCM not being concentrated using centrifugal filters in this study prior to precipitation. The frozen aliquot of 20 mL of CCM was thawed at room temperature on the day of use and processed immediately once liquid, while still cold. The CCM was then mixed with 0.5 volume of Total Exosome Isolation Reagent (i.e., 10 mL) (Invitrogen, Cat#4478359) and vortexed for 30 s to 1 min at maximum speed until the solution was homogenous. The sample was incubated overnight at 4 °C and was centrifuged at 10,000×*g* for 1 h at 4 °C the next day. The supernatant was removed and the EV pellet was suspended in 1 mL of filtered D-PBS^−/−^. For this study, the 20-mL samples (EV-rich CCM sample) were collected daily; however, only the samples harvested at collection days 1–2 (start), days 13–14 (interim), and days 24–25 (end) were used for downstream analyses of EVs.

### Nanoparticle tracking analysis (NTA) of EVs using the NanoSight NS300

EVs were analyzed by nanoparticle tracking analysis (NTA) using the NanoSight NS300 (Malvern Panalytical), as previously described by our group with slight modifications [6]. Twenty milliliters of EV-rich CCM was used. Following EV purification as described in the “EV purification from the EV-rich hBM-MSC-conditioned medium collected from the hollow-fiber bioreactor system” section, the EV pellet was suspended in 1 mL of filtered D-PBS^−/−^. Hundred microliters of the 1-mL EV-rich sample was used for NTA and diluted 10–40× in filtered D-PBS^−/−^ to obtain a final volume of 1 mL of EV sample for analysis. Each sample was vortexed prior to filling the syringe with the sample and the syringe pump from Harvard Apparatus (cat.# 98-4730) was used for acquisition in flow mode. Each 1-mL sample was run using the following script: six captures of 1 min at speed 10 under flow mode. For capture settings, a camera level of 15 was used for all samples and a detection threshold of 30 was used for analysis resulting in approximately 30 particles per frame. Analysis of the raw data was performed using the NTA 3.0 software (Malvern Instruments) where analysis of five out of six captures was performed, removing the first capture to generate the approximate total EV concentration. Following NTA data analysis using the NTA 3.0 software, Excel was used to account for the dilution factor 10.

### Lectin microarray analysis of EVs

The EV samples used for the lectin microarray analysis were the same as the ones prepared for the NTA analysis described in the “Nanoparticle tracking analysis (NTA) of EVs using the NanoSight NS300” section. From the 1-mL EV-rich sample, an EV aliquot corresponding to 10 μg of EVs (measured by absorbance at 280 nm using NanoDrop One, ThermoFisher Scientific) was diluted to 100 μL with filtered D-PBS^−/−^ and was used for the glycan sample preparation for each of the samples (*N* = 4 donors at the 3 time points of collection). Results of protein quantification are provided in Supplementary Table 2. Each EV sample was labeled overnight at 4 °C with 1 μL of DyLight550 NHS Ester (ThermoFisher Scientific, cat.#62262). Filtered PBS treated with DyLight550-NHS was used as a negative control. Excess dye was quenched with 50 μL of 250 mM Tris buffer pH 8.0 for 30 min at room temperature, for a final concentration of 66.7 μg/mL for each EV sample.

The arrayed glass slide containing 26 lectins printed in triplicates (Lectin array, 16 subarray cassettes, ZBiotech, Aurora, cat.#10605) was incubated on a shaker at 85 rpm for 1 h with 100 μL blocking buffer (1% BSA in PBS, 0.05% v/v Tween-20, pH 7.4) per well. After blocking, wells were rinsed with assay buffer (ZBiotech) and the DyLight550-labeled EVs were applied (100 μL/well). The lectin slide was covered in foil and incubated overnight on a shaker (85 rpm, room temperature). Samples were removed from all wells and slides were washed 2 times with 150 μL of wash buffer (20 mM Tris, 150 mM NaCl, 0.05% Tween-20, pH 7.6) followed by a soak in wash buffer for 10 min on a shaker then a soak in water for 5 min. The slide was dried by centrifugation at 200 rpm for 1 min, then scanned using the Genepix 4000B (Molecular Devices). Fluorescence was measured at 532 nm with PMT gain at 400 and 100% power. Fluorescence intensity of each sample spot was corrected by subtracting the background signal (intensity of the same lectin spots in a negative control well) and the average of the triplicate lectin fluorescent intensities in each well was calculated.

### EV glycan sample preparation for capillary electrophoresis-laser-induced fluorescence (CE-LIF) analysis

The EV samples used for the capillary electrophoresis-laser-induced fluorescence (CE-LIF) analysis were the same as the ones prepared for the NTA analysis described in the “Nanoparticle tracking analysis (NTA) of EVs using the NanoSight NS300” section. From the 1-mL EV sample, a 20-μL aliquot was used for the EV glycan sample preparation. This 20-μL EV aliquot was dried down in a vacuum concentrator and resuspended in 10 μL of double deionized water (ddiH_2_O). The EV samples were processed using the Sciex Fast Glycan Labeling and Analysis Kit (Sciex, cat.#B94499) for denaturation, enzymatic glycan release (PNGase F, 500 NEB units) (New England Biolabs, cat.#P0704), fluorophore labeling (8-aminopyrene-1,3,6-trisulfonate; APTS) (Sciex, cat.#B94507), and excess dye removal by magnetic beads. The CE-LIF analysis was performed using a Beckman Coulter (Sciex) PA800 Plus Pharmaceutical Analysis System equipped with a solid state laser-induced fluorescence detector (*λ*_ex_ = 488 nm/*λ*_em_ = 520 nm). CE consumables were purchased from Sciex. Analytical reagent grade acetonitrile (ACN) and 2-propanol (IPA) were purchased from Merck KGaA. Ammonium acetate and acetic acid were purchased from Sigma-Aldrich. All separations were carried out using a background electrolyte (BGE) consisting of 7.5 mM ammonium acetate pH 4.5, 10% isopropanol in a 50-cm effective length (60 cm total length), 50 μm ID polyvinyl alcohol (PVA)-coated capillary. The separation voltage was set to 30 kV in reversed polarity mode (cathode at injection side) and the separation temperature was set to 20 °C. Sample injection was performed hydrodynamically by applying 0.5pSi forward pressure for 10 s (~ 9 nL). 32Karat (Sciex, version 10.1) was used for data acquisition and processing. The traces for comparison of the glycan profiles were normalized to the internal standard (maltotriose) and *y*-axes were scaled to account for the concentration differences obtained by NTA.

### Immunophenotyping of EVs using the MACSplex Exosome kit of 37 specific markers

The MACSplex Exosome kit (i.e., multiplex bead-based flow cytometric analysis) was conducted as previously described by our group with slight modifications [6]. Twenty milliliters of the EV-rich CCM harvested as described in the “EV purification from the EV-rich hBM-MSC-conditioned medium collected from the hollow-fiber bioreactor system” section was assessed using the MACSplex Exosome kit (human) (Miltenyi Biotec, cat.#130-108-813), where the EV pellet was suspended in 1mL of filtered PBS. Following isolation, 460 μL of EV samples (out of 1 mL) were transferred to 1.5-mL Protein LoBind tubes (Eppendorf, cat.#0030.108.116) where 40 μL of MACSplex Exosomes Capture Beads was added to each EV sample and incubated overnight. Samples were processed as per the manufacturer’s recommendations using the “overnight protocol for the assay using 1.5mL tubes” and detection of EVs was done using the CD63 MACSplex Exosome Detection Reagent. Following labeling, samples were transferred to 5-mL FACS tubes (BD Biosciences, cat.#382058) and analyzed by flow cytometry using the LSRII flow cytometer (BD Biosciences). Ten thousand events per sample were collected. Raw data was analyzed using FlowJo V10 (FlowJo LLC, USA). For data processing, buffer only control for each specific bead was used for background subtraction for the respective bead population. Following buffer subtraction, the IgG isotype control signal was subtracted from all bead populations. Finally, the detection threshold of MFI > 20 was set based on the residual signal present in the negative channels.

### Cytokine/chemokine/growth factor analysis of the EV protein cargo using the cytokine/chemokine 29-plex magnetic bead panel kit

Five milliliters of the EV-rich CCM harvested as described in the “EV purification from the EV-rich hBM-MSC-conditioned medium collected from the hollow-fiber bioreactor system” section was assessed for the detection of cytokine/chemokine/growth factors using the human cytokine/chemokine 29-plex magnetic bead panel kit (Millipore Sigma, cat.# HCYTMAG-60 K-PX29). Once EVs were precipitated, the pellet was suspended in 0.2 mL of lysis buffer (200 μl PBS + 0.2% Triton X-100 + 2.0 μl of HALT protease and phosphatase inhibitors 100X) (HALT Protease and Phosphatase Inhibitors 100X; Thermofisher Cat # PI78441) and transferred to a 1.5 mL Protein LoBind tube (Eppendorf, cat.#0030.108.116). Each sample was then put on an end-over-end shaker (LabQuake Shaker) and incubated for 30 min at 4 °C. The sample was then spun down quickly to collect the EV lysate before sonicating (amplitude setting at 20%, pulsed for 10 s) and put on ice for 30 s. This was repeated 3 times. The EV protein lysate was centrifuged at 14,000×*g* for 5 min at 4 °C. The supernatant was recovered and stored in LoBind tubes at − 80 °C. Protein concentration was measured using the BCA Protein Assay Kit (Thermofisher, cat.# PI23227) for normalization purposes using total protein content. For the cytokine detection, each EV lysate was thawed on ice and 25 μl of EV lyzed protein sample was used for analysis. Samples were processed as per the manufacturer’s protocol for the cytokine/chemokine 29-plex magnetic bead panel kit and analyzed on the Luminex platform (Luminex 100/200 System, Luminex Corp.). Quality control for the fit of each 4 log standard curve for each cytokine was set with a cut-off of chi < 1%. Values below the detection threshold of the standard curve for each cytokine were presumed to be zero. For data processing, the lysis buffer only was added as a control for background subtraction; however, no signal was detected for any of the cytokines analyzed based off this control. All samples were measured in duplicate and the average was used for analysis. The cytokine concentration obtained in pg/mL was then normalized to the total protein extract data for each sample measured by BCA (i.e., pg/mL/μg of total protein extract).

### Statistical analysis

Biological (hBM-MSC donors #48RB, #81RB, #55RB, and #85RB) and technical replicates were used and are indicated in figure legends. Statistical significance was analyzed by a two-way analysis of variance (ANOVA) with Tukey’s post hoc analysis as indicated in the figure legends. Statistical analyses were performed using GraphPad Prism version 6.0 (GraphPad Software Inc., La Jolla, CA, USA). A *p* value of < 0.05 was considered statistically significant and significance differences are marked with a single (*p* < 0.05), double (*p* < 0.01), triplicate (*p* < 0.001), or a quadruple (< 0.0001) asterisk.

## Results

### Culture-expanded hBM-MSCs showed characteristics suitable for hollow-fiber cell bioreactor inoculation

**Fig. 1:**
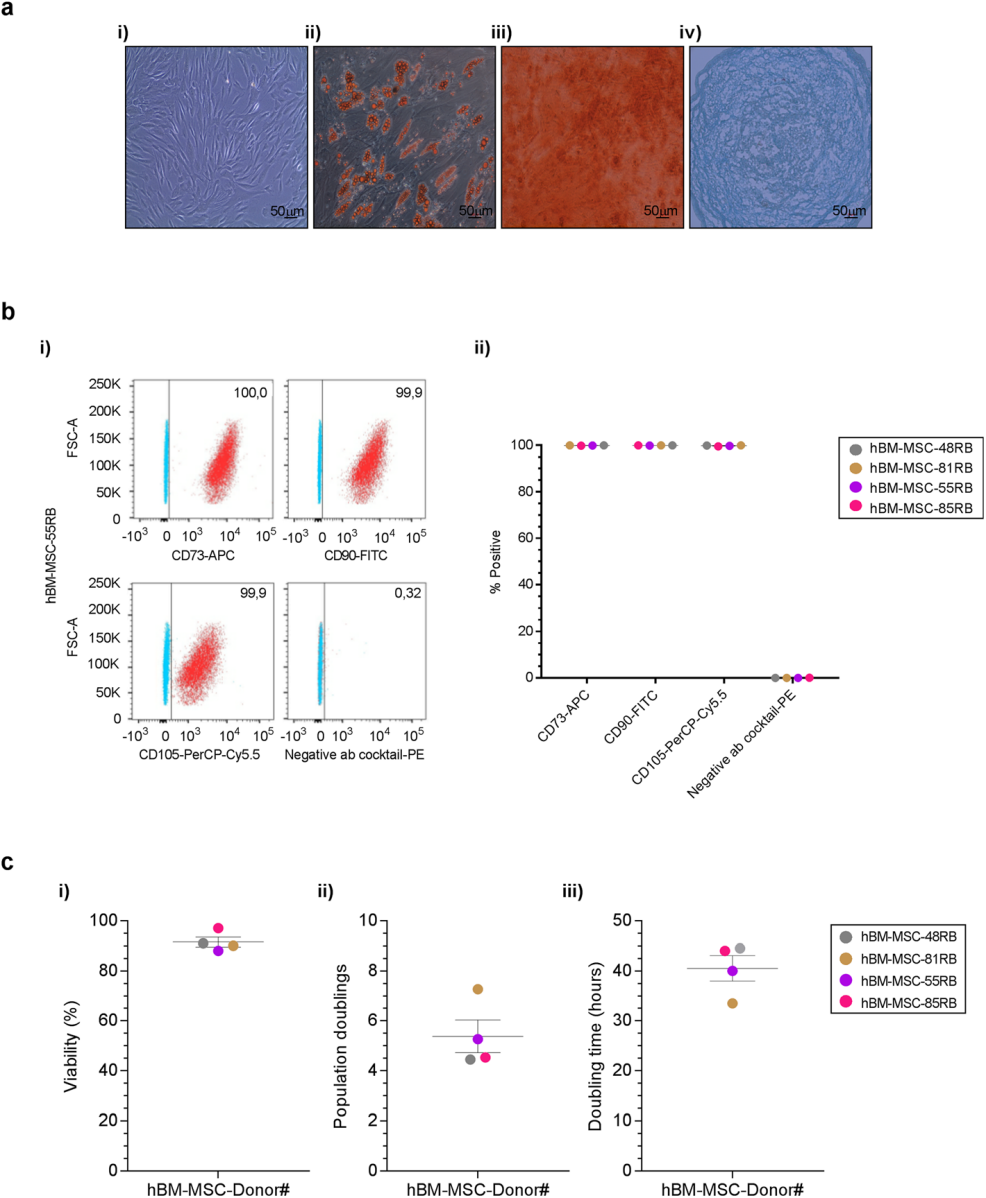
Assessment of hBM-MSC characteristics after CellSTACK cell culture expansion confirms cell identify. **a** Representative microscopic images of undifferentiated hBM-MSCs (i) or chemically stained for detection of adipocytes (ii), osteocytes (iii), and chondrocytes (iv) following induction of differentiation of the cells into the three-mesoderm lineages. All images were taken using a × 20 objective (FOV of 0.43) and the Zeiss Axio Observer 5 microscope. Adipocytes were stained with Oil Red O, osteocytes stained with Alizarin S, and chondrocytes stained with Alcian Blue. Each hBM-MSC donor (*N* = 4 donors; hBM-MSC-48RB/81RB/55RB/85RB) was assessed for differentiation potential. **b** Flow cytometric analysis of established positive and negative MSC surface markers. Red indicates the cell population stained with the respective antibodies for the detection of positive and negative antigens. Blue indicates the cells stained with an IgG isotype control. Negative antibody (ab) cocktail indicates cells stained with a mixed cocktail of antibodies for the detection of MSC negative markers: HLA-DR, CD11b, CD19, CD34, and CD45 (i). Quantification of percentage of positive cells analyzed by flow cytometry (ii). **c** Viability analysis (i) of each hBM-MSC donor (*N* = 4 donors; hBM-MSC-48RB/81RB/55RB/85RB) recovered from the CellSTACK culture expansion system prior to inoculation into the bioreactor system. Percentage of viable cells were generated by automated counting of dye negative over dye positive cell populations stained using Trypan Blue (*n* = 2 technical replicates per donor). Population doublings (ii) and doubling time (iii) of each hBM-MSC donor (*N* = 4 donors; hBM-MSC-48RB/81RB/55RB/85RB) calculated over 4 days of expansion in the CellSTACK culture expansion system (*n* = 2 technical replicates per donor)

Working cell banks were created from pre-characterized bone marrow mesenchymal stromal cells (hBM-MSCs) obtained from RoosterBio Inc. (Supplementary Table 1) [6]. Human BM-MSCs from four different healthy male (18–41 years old) bone marrow donors (hBM-MSC-48RB, -81RB, -55RB, -85RB) were characterized at passages 2–3 according to the minimal criteria of the International Society of Gene and Cellular Therapy (ISCT) [26]. The hBM-MSCs maintained plastic adherence (Fig. 1a (i)) and upon induction of trilineage mesoderm differentiation, the cells from the four donors displayed differentiation capacity towards adipogenesis (Fig. 1a (ii)), osteogenesis (Fig. 1a (iii)), and chondrogenesis (Fig. 1a (iv)). The hBM-MSCs portrayed expected expression of positive (> 95% for CD73, CD90, and CD105) and negative (< 2% of the negative antibody cocktail for CD11b, CD19, CD34, CD45, HLA-DR) MSC-known markers (Fig. 1b and Supplementary Table 1). Importantly, the cells were expanded in the CellSTACK cell culture chambers to reach high cell numbers (90–220 million), required, for seeding the cartridge of the hollow-fiber cell bioreactor system. During this process, the hBM-MSCs from the four donors retained high viability (92 ± 2%, mean ± S.E.M.) (Fig. 1c (i)) and showed a population doubling of 5.4 ± 0.7 (mean ± S.E.M.) (Fig. 1c (ii)) and doubling time of 40.5 ± 2.5 h (mean ± S.E.M.) (Fig. 1c (iii)).

### The hollow-fiber cell bioreactor system sustained cell health and functionality of hBM-MSCs over the inoculation period

**Fig. 2:**
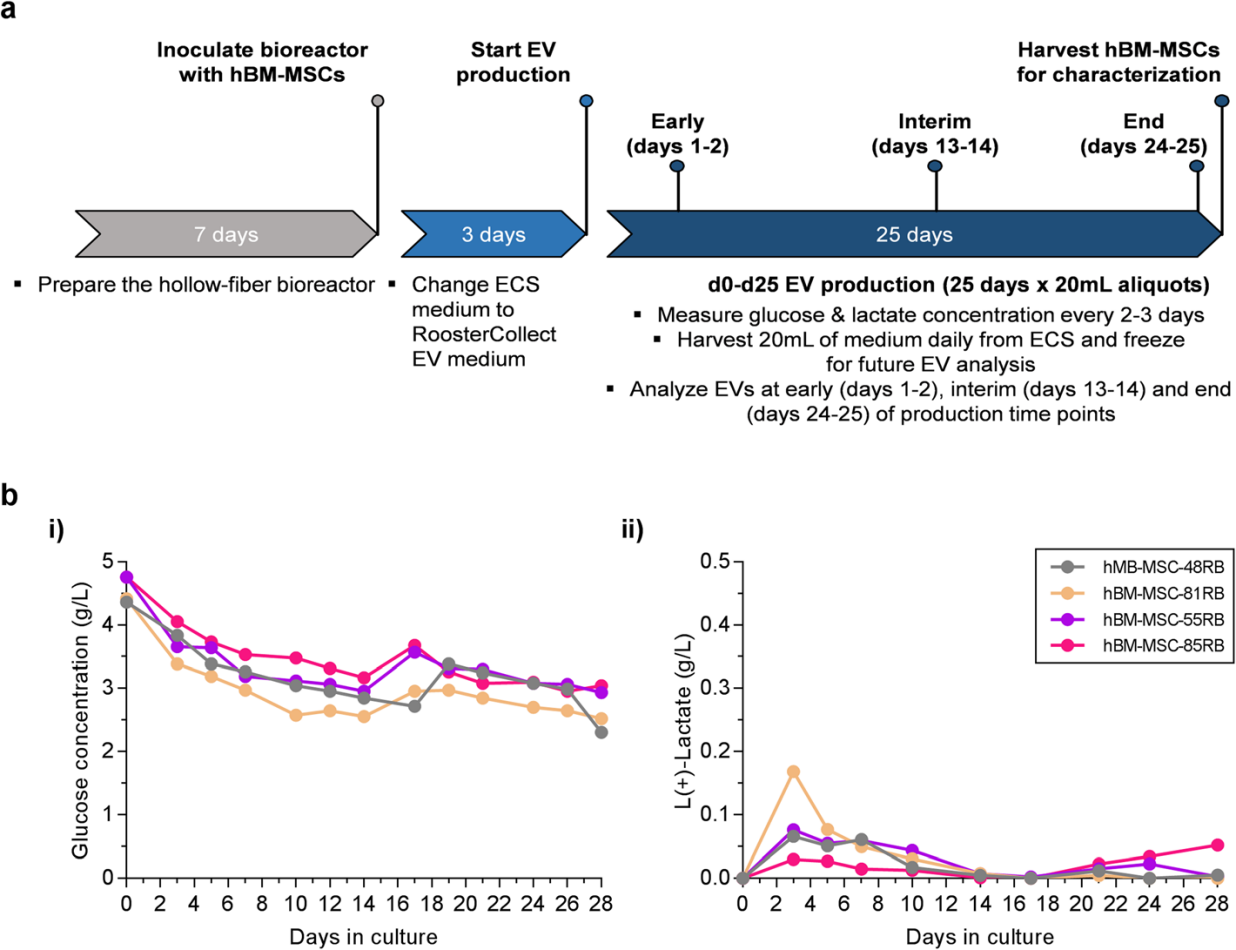
Monitoring of culture parameters during the hollow-fiber bioreactor incubation period demonstrates feasibility and consistency of cell homeostasis. **a** Graphical schematic representation of the entire bioreactor-based workflow for the production of extracellular vesicles from hBM-MSCs in the hollow-fiber bioreactor system. A 7-day preparation period of the hollow-fiber cell culture system was required before cell inoculation where PBS was injected for 5 days followed by a 2-day incubation with RoosterBio complete cell culture medium. At day 7, hBM-MSCs were inoculated into the hollow-fiber cartridge. The medium in the extracellular capillary space (ECS) was replaced by the RoosterCollect-EV medium for 2 days during this 3-day period of cell inoculation. At day 10, the 25-day EV production period started and 20 mL of the EV-rich cell-conditioned medium was retrieved daily from the ECS and frozen for future EV isolation and analysis. Aliquots of EV-rich cell-conditioned medium harvested at early (days 1–2), interim (days 13–14), and end (days 24–25) of production time points were selected for further EV downstream analysis. The glucose and lactate concentrations were measured every 2–3 days. At the end of the 25-day EV production period, hBM-MSCs were harvested from the cartridge and analyzed for MSC features. **b** Glucose concentration (i) in the circulating medium was monitored at a 2–3-day interval schedule to ensure consistent glucose consumption by cells. Indicated by the inflections between days 16 and 18 is representative of glucose replenishment by adding fresh cell culture medium. L-Lactate production was assessed using circulating media samples by measuring L-Lactate concentrations (ii) using a colorimetric plate-based assay to ensure cells did not achieve metabolic stress (*N* = 4 donors; hBM-MSC-48RB/81RB/55RB/85RB) (*N* = 2 technical replicates)

CellSTACK culture-expanded hBM-MSCs were inoculated into the hollow-fiber cell bioreactor system to allow for a continuous production of EVs for 25 days (Fig. 2a). Twenty milliliters of the EV-rich cell-conditioned medium was retrieved daily from the extracapillary space (ECS) and frozen for future EV isolation and analysis. Aliquots of EV-rich cell-conditioned medium harvested at early (days 1–2), interim (days 13–14), and end (days 24–25) of production time points were selected for further EV downstream analysis (Fig. 2a). Ensuring that the hBM-MSCs maintained their viability during the 25-day EV production timeline is a vital component of producing EVs of high quality with the expected physio-chemical properties. During the entire cell incubation period of 28 days into the hollow-fiber bioreactor system, hBM-MSCs from the four donors consistently consumed glucose (Fig. 2b (i)). Noteworthy, the fresh culture medium was added to the system between day 14 and 17 when the total glucose concentration reached approximately 58–66% of the starting amount (4.6 ± 0.1 g/L, mean ± S.E.M.). Lactate concentration readings showed that the cells were not producing elevated amounts of L-lactate (< 0.2 g/L), as expected (Fig. 2b (ii)). Furthermore, pH was measured every 2–3 days and remained between 7.0 and 7.4 throughout the 28-day hBM-MSC incubation period (data not shown), highlighting that pH stability was preserved during cell incubation in the bioreactor. These results show that cell health and homeostasis measured by surrogate markers (glucose consumption, lactate production, and pH) were maintained throughout the cell incubation period in the bioreactor.

### hBM-MSCs harvested from the hollow-fiber cell bioreactor system retained their trilineage mesoderm differentiation capacity and showed plasticity towards surface marker expression profile

**Fig. 3:**
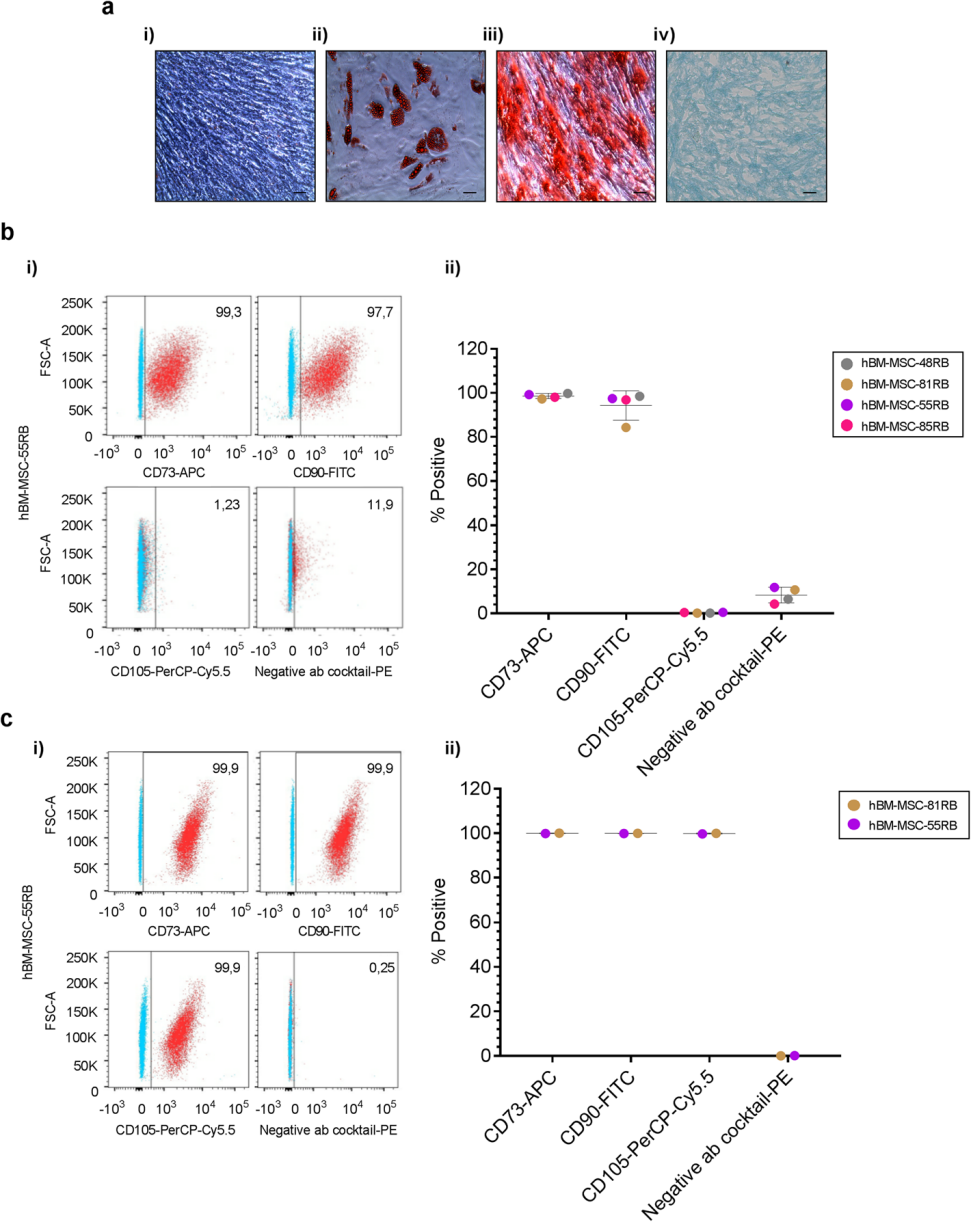
Post-bioreactor analysis of hBM-MSCs confirms retention of MSC characteristics. **a** Representative microscopic images of undifferentiated hBM-MSCs (i) or chemically stained for detection of adipocytes (ii), osteocytes (iii), and chondrocytes (iv) following induction of differentiation of the cells into the three-mesoderm lineages. All images were taken using a × 20 objective (FOV of 0.43) and the Zeiss Axio Observer 5 microscope. Adipocytes were stained with Oil Red O, osteocytes stained with Alizarin S, and chondrocytes stained with Alcian Blue. Each hBM-MSC donor (*N* = 4 donors; hBM-MSC-48RB/81RB/55RB/85RB) was assessed for differentiation. **b** Flow cytometric analysis of established MSC surface markers following immediate recovery of cells from the hollow-fiber bioreactor system (*N* = 4 donors; hBM-MSC-48RB/81RB/55RB/85RB). Red indicates the population stained with the respective antibodies for the detection of positive and negative markers. Blue indicates the cells stained with an IgG isotype control. Negative antibody (ab) cocktail indicates cells stained with a mixed cocktail of antibodies for the detection of MSC negative markers: HLA-DR, CD11b, CD19, CD34, and CD45 (i). Quantification of percentage of positive cells analyzed by flow cytometry for the expression of markers from hBM-MSCs post-bioreactor recovery (ii). **c** Flow cytometric analysis of established MSC surface markers was performed following 7 days of recovery of cells in 2D flask-based culture system using normal RoosterBio culture medium. Representative flow cytometric plots (i) and quantification of the flow cytometric results (ii) of hBM-MSC #55RB and #81RB donors. Red indicates the population stained with the respective antibodies for the detection of positive and negative markers. Blue indicates the cells stained with an IgG isotype control. Negative antibody (ab) cocktail indicates cells stained with a mixed cocktail of antibodies for the detection of MSC negative markers: HLA-DR, CD11b, CD19, CD34, and CD45

Human BM-MSCs were retrieved at the end of their 28-day incubation period into the bioreactor and assess for mesoderm trilineage differentiation to test whether they retained this characteristic. Bioreactor-harvested hBM-MSCs showed high viability (> 85%) (data not shown), and upon incubation into an inductive medium for trilineage mesoderm differentiation, the cells from the four donors (Fig. 3a (i)) showed a differentiation capacity towards adipogenesis (Fig. 3a (ii)), osteogenesis (Fig. 3a (iii)), and chondrogenesis (Fig. 3a (iv)). To test whether the bioreactor culture conditions altered the immunophenotypic characteristics of the seeded hBM-MSCs, assessment of MSC-known surface markers was performed by flow cytometry (Fig. 3b (i)). Interestingly, harvested hBM-MSCs retained a high expression of CD73 (98.6 ± 0.6%, mean ± S.E.M) and CD90 (94.3 ± 3.3%, mean ± S.E.M) MSC-positive markers, while showing a marked decreased expression of CD105 (0.3 ± 0.1%, mean ± S.E.M) levels (Fig. 3b (ii)). In contrast, the expression of the negative antibody cocktail (antibodies against HLA-DR, CD11b, CD19, CD34, CD45 antigens) showed a low positive signal of 8.3 ± 1.8% (mean ± S.E.M) as an averaged for the four donors (Fig. 3b (ii)). To determine which antigen from the negative antibody cocktail contributed to the positive staining from the bioreactor-harvested hBM-MSCs, HLA-DR and CD34 antibodies were selected to be tested separately, as the other 3 antigens were determined unlikely to contribute to the positive signal. Importantly, HLA-DR antigen expression level showed less than 0.3% of positive (0.3 ± 0.1%, mean ± S.E.M) in contrast to the CD34 antigen expression level which showed 6.7 ± 1.7% (mean ± S.E.M) positive (Supplementary Figure 1). Furthermore, to understand the absence of CD105 antigen and the presence of a low positive signal arising from the CD34 antigen, bioreactor-retrieved hBM-MSCs from donor 55RB and 81RB were seeded back into the normal medium conditions (RoosterBio medium) for 7 days after which time cells were re-assessed by flow cytometry for the same panel of antigens (Fig. 3c (i)). As expected, flow cytometric analysis showed a high expression level of CD73 (99.9 ± 0.1%, mean ± S.D.) and CD90 (99.9 ± 0.1%, mean ± S.D.) antigens (Fig. 3c (ii)). Remarkably, the CD105 expression level was recovered from the two donors tested (hBM-MSC-55RB and -81RB) following the 7-day incubation in the normal cell culture medium where 99.9 ± 0.2% (mean ± S.D.) of the cells expressed CD105. These results showed that the bioreactor-retrieved hBM-MSCs retained the ability to express CD105 at their membrane surface level once seeded under normal culture medium conditions. Interestingly, the hBM-MSCs recovered in the normal cell culture medium from the two donors tested also showed a low expression level of the negative cocktail antibody (0.10 ± 0.08%, mean ± S.D.). Overall, these combined results showed that the hBM-MSCs harvested from the bioreactor retained MSC functionality and immunophenotypic characteristics.

### EVs from hBM-MSCs maintained a small EV size profile with a consistent glycan signature throughout the 25-day production period in the hollow-fiber cell bioreactor system

**Fig. 4:**
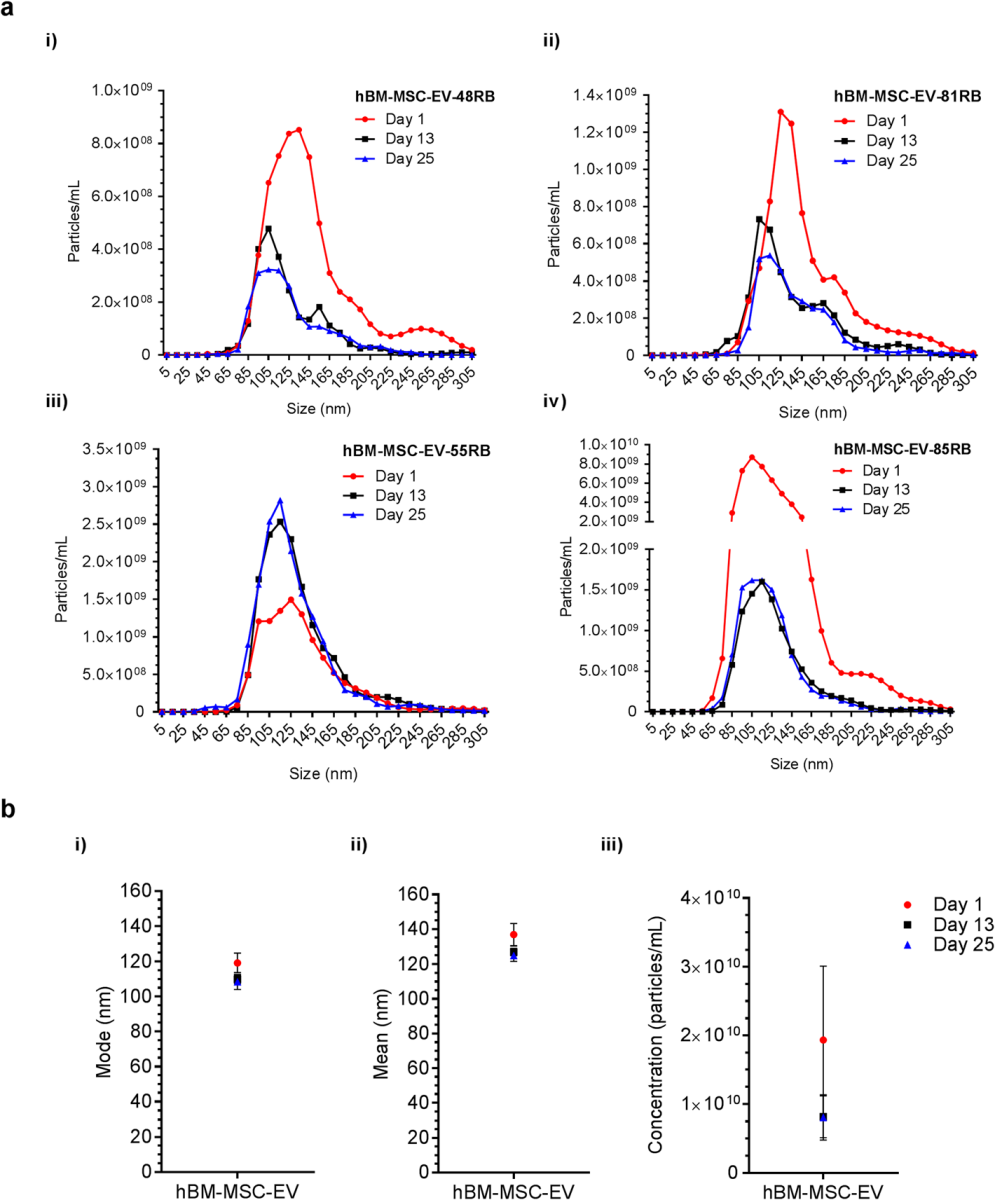
EV production from hBM-MSCs in the hollow-fiber cell bioreactor system yields nanovesicles of small EV distribution profile. **a** Histogram plots of nanoparticle size concentration and distribution of the four hBM-MSC donors (*N* = 4 donors; hBM-MSC-48RB/81RB/55RB/85RB donors) at 3 different time points (days 1, 3, and 25) of EV production (i–iv). Red represents EV material processed at day 1, black represents EV material processed at day 13, and blue represents the final EV product processed at day 25. The mean for each dot represents 5 technical replicates from each hBM-MSC donor. All data was captured on the NanoSight NS300 (Malvern Panalytical) on flow mode. **b** The EV mode (nm) (i), mean (nm) (ii), and concentration (particles/mL) (iii) are represented as an averaged of the four hBM-MSC donors at each time points

During the 25-day EV production timeline in the hollow-fiber cell bioreactor system, a 20-mL EV-rich cell-conditioned medium (CCM) sample was collected daily and assayed for downstream EV purification and analysis. EV samples collected at the start (day 1), interim (day 13), and end (day 25) of the EV production period were assessed by Nanoparticle Tracking Analysis (NTA) for particle size distribution and concentration (Fig. 4 and Supplementary Table 3). Figure 4a shows the individual EV particle size distribution and concentration plots for each of the four hBM-MSC donors analyzed at the three collection time points (days 1, 13, and 25) (Fig. 4a (i–iv)). Particle size analysis of EV samples from the four hBM-MSC donors showed a mode ranging from 103 to 128 nm at day 1, 102 to 114 nm at day 13, and 96 to 116 nm at day 25 (Supplementary Figure 2 and Supplementary Table 3), fitting the small EV size category (50–200 nm) described in the ISEV guidelines [28]. When averaged, the four hBM-MSC donors presented a mode size of 119 ± 6 nm at day 1, 110 ± 2 nm at day 13, and 108 ± 4 nm at day 25 (Fig. 4b (i)). When averaged, the four hBM-MSC donors presented a mean size of 137 ± 6 nm at day 1, 127 ± 2 nm at day 13, and 125 ± 3 nm at day 25 (Fig. 4b (ii)). Additionally, semi-quantitative analysis of EV concentrations ranged from 6.7 × 10^9^ to 5.2 × 10^10^ particles/mL at day 1, 2.5 × 10^9^ to 1.6 × 10^10^ particles/mL at day 13, and 2.2 × 10^9^ to 1.6 × 10^10^ particles/mL at day 25 for the four donors (Supplementary Figure 2 and Supplementary Table 3). When averaged, the four hBM-MSC donors presented a concentration of 1.9 × 10^10^ ± 1.1 × 10^10^ particles/mL at day 1, 8.2 × 10^9^ ± 3.0 × 10^9^ particles/mL at day 13, and 8.1 × 10^9^ ± 3.3 × 10^9^ particles/mL at day 25 (Fig. 4b (iii)). To verify whether pooling harvested EV-rich CCM samples would yield an EV sample of similar nanoparticle size distribution, 5-mL aliquots of EV-rich CCM harvested each day from the hollow-fiber system were pooled from days 1 to 25 (hBM-MSC-81RB donor, samples obtained from a duplicate hollow-fiber bioreactor run), resulting in a 125-mL bulk sample. Switching from a low to a bulk volume of sample to process, tangential flow filtration coupled with fast protein liquid chromatography (FPLC) was employed to purify the EV sample. The FPLC-purified pooled EV sample yielded a comparable size distribution result as the TEI-precipitated EV sample (< 200 nm in size with a mode of 110.6 nm and mean of 114.1 nm) (Supplementary Figure 3). Furthermore, transmission electron microscopy (TEM) further corroborated the small EV size category showing nanoparticles with a size of less than 200 nm (Supplementary Figure 4).

**Fig. 5:**
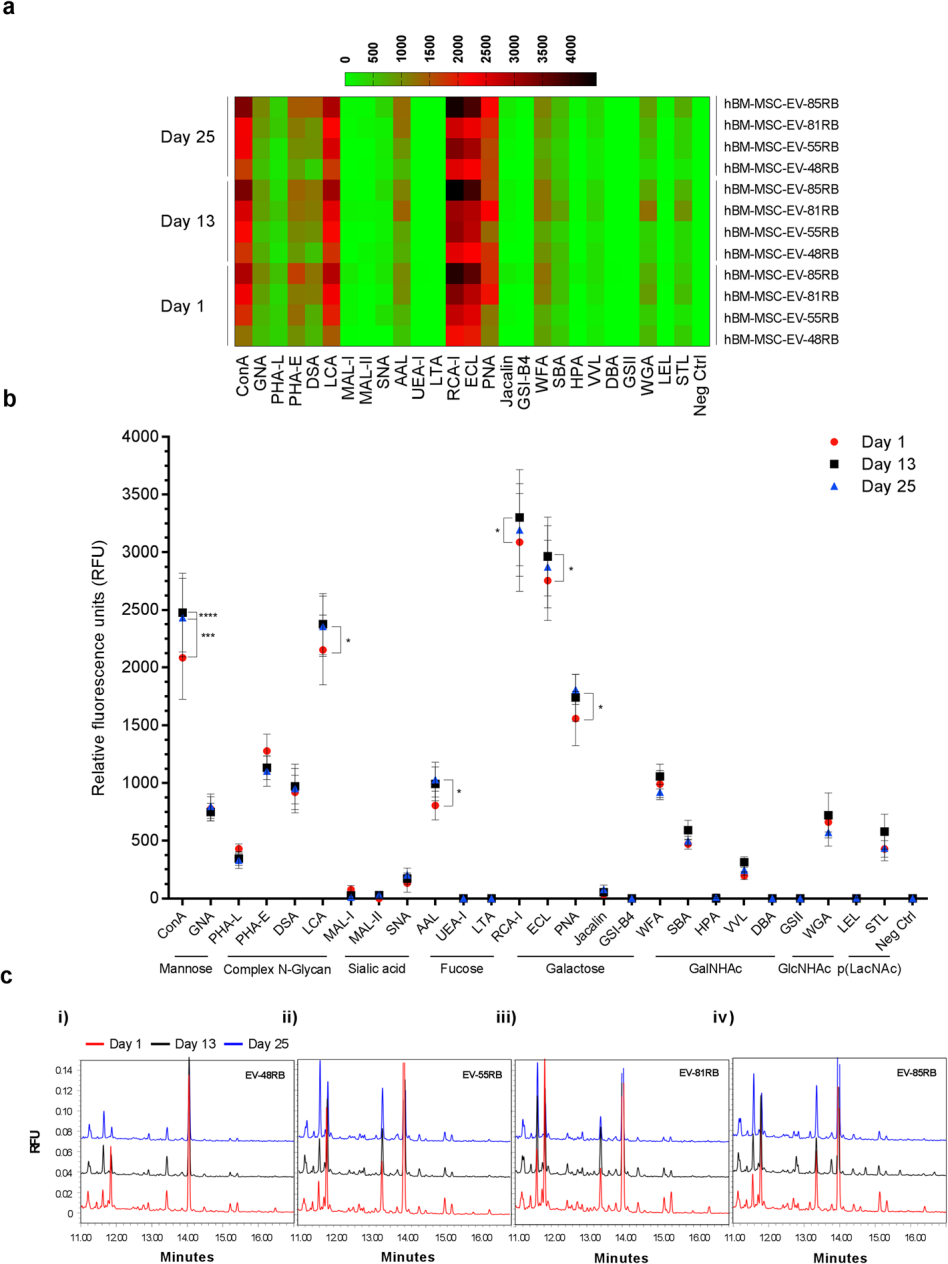
EVs produced in the hollow-fiber system from hBM-MSCs produce consistent glycan profiles. Glycan analysis of EVs isolated from each hBM-MSC donor (*N* = 4 donors; hBM-MSC-48RB/55RB/81RB/85RB) over several days (days 1, 13, and 25) confirms consistent glycoprofiles of EVs. **a**, **b** Lectin microarray analysis of EVs (66.7 μg/mL) labeled with DyLight-555-NHS. **a** Heat map showing the relative intensity of fluorescently labeled EVs, from four individual hBM-MSC donors at three collection time points, bound to 26 glycan-binding lectins on a microarray slide (Z-Biotech). **b** Quantitative analysis of the lectin microarray fluorescence signal of the mean of the four hBM-MSC donors at the three collection time points is represented. Each dot represents *N* = 4 donors, each analyzed in triplicates. Statistical significance was analyzed by a two-way analysis of variance (ANOVA) with Tukey’s post hoc analysis. A *p* value of < 0.05 was considered statistically significant and significant differences are marked with a single (*p* < 0.05), triple (*p* < 0.001), or quadruple (*p* < 0.0001) asterisks. **c** Qualitative comparison of the capillary electrophoresis-laser-induced fluorescence (CE-LIF) traces from enzymatically released and APTS-labeled glycan profile from EV samples collected at three collection time points during EV production from four hBM-MSC donors. The traces for the glycan profile for the EV samples harvested at day 1 (red), day 13 (black), and day 25 (blue) are displayed for donors hBM-MSC-48RB (i), hBM-MSC-55RB (ii), hBM-MSC-81RB (iii), and hBM-MSC-85RB (iv), respectively

To further analyze the time-dependent quality of EV production in the hollow-fiber cell system, we analyzed the composition of glycans of the EVs. Glycan composition is a post-translational modification that is dictated by intra- and extracellular glycosyl transferases and glycosidases, and is therefore sensitive to changes in biotherapeutics manufacturing environments [29], thereby rendering it an indicator of consistency in the product manufacturing process. To analyze the glycan signature on the surface of EVs, we performed lectin microarray analysis, which revealed generally consistent time-dependent expression of specific lectin-binding glycans for each of the four donors (Fig. 5). In particular, glycans that bound to RCA-I/ECL/PNA (galactose-bearing glycans), PHA-E/DSA/LCA (complex N-glycans), and ConA (mannose glycans) lectins were consistently expressed among all individual donors at days 1, 13, and 25 (Fig. 5a), with RCA-I, ECL, PNA, LCA, and ConA-binding glycans yielding the highest signal intensities (> 1500 RFU). Quantitative analysis showed that glycans that bound ConA and LCA were slightly, but statistically significantly higher on days 13 and 25 vs day 1 (*p* < 0.05), while RCA-I and ECL-binding glycans were higher on day 13 than day 1 (*p* < 0.05), and AAL and PNA-binding glycans were higher on day 25 vs day 1 (*p* < 0.05) (Fig. 5b). We further assessed the *N*-glycan signature on EVs by capillary electrophoresis with laser-induced fluorescence detection (Fig. 5c). Comparison of the enzymatically released *N*-glycan profiles of EVs obtained from days 1, 13, and 25 from the four hBM-MSC donors showed no observable changes in the *N*-glycan profiles, further indicating that the chemical consistency of the EV material in production was maintained (Fig. 5c).

### EVs produced by hBM-MSCs inoculated in the hollow-fiber cell bioreactor system harbor expressional signatures reflective of the immuno-modulatory properties of the producer cell

**Fig. 6:**
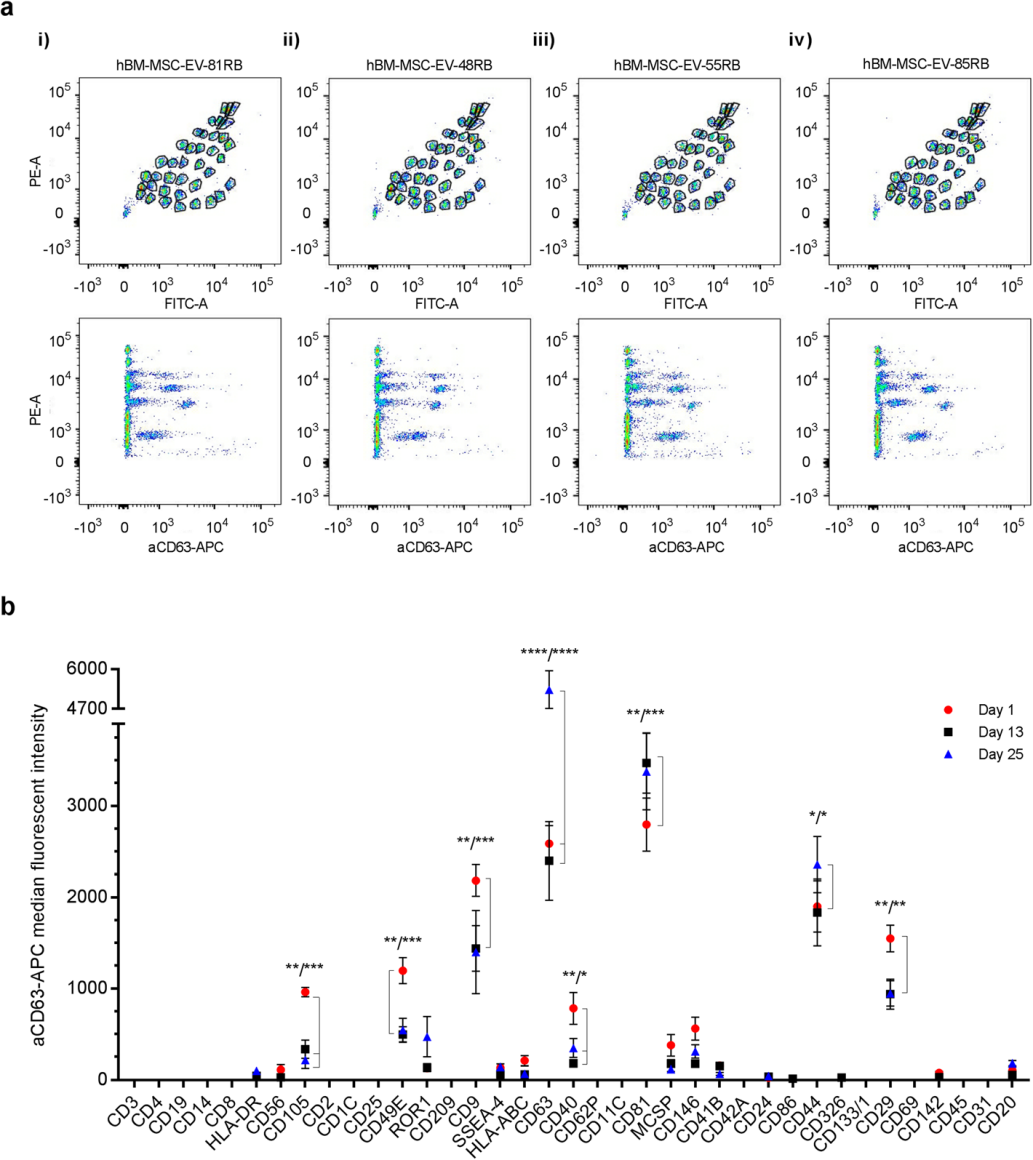
Multiplex antibody bead-based surface profiling analysis of EVs reveals an immuno-modulatory signature. **a** Representative flow cytometric dot plots of 37 surface epitopes plus two isotype controls (39-bead plex) are shown for each of the four hBM-MSC donors (81RB/48RB/55RB/85RB; panels i–iv, respectively). The flow cytometric plots represent the 39-bead gating areas representative of each epitope analyzed with its own gate according to the calibration bead distribution (top panels). Positive EV populations were detected using a CD63-APC detection antibody (lower panels) and double positive (specific epitope: CD63-APC) populations are shown in the multiplex analysis. **b** Quantification of the flow cytometric results obtained by the MACSPlex multiplex analysis for each of the EV samples harvested at three time points of collection (days 1, 13, and 25) and of all four hBM-MSC donors (*N* = 4; hBM-MSC-EV-81RB/48RB/55RB/85RB). Data are showing the mean for each time point of collection and standard error of mean (S.E.M) represent the four hBM-MSCs. Statistical significance was analyzed by a two-way analysis of variance (ANOVA) with Tukey’s post hoc analysis. A *p* value of < 0.05 was considered statistically significant and significant differences are marked with a single (*p* < 0.05), double (*p* < 0.01), triple (*p* < 0.001), or quadruple (*p* < 0.0001) asterisks

A repertoire of 37 epitopes targeting EV-, stem cell-, and immune-related antigens was assessed using a multiplex bead-based flow cytometric assay where EV samples collected at days 1, 13, and 25 were interrogated for expression levels (Fig. 6 and Supplementary Table 4). Representative flow cytometric plots of the 39-bead population (37-specific antigens and 2 isotype controls) detected by the anti-CD63-APC reagent generated at day 1 are shown in Fig. 6a for each hBM-MSC donor (Fig. 6a (i–iv)). Tetraspanins CD9, CD63, and CD81 were highly detectable (> 1000 median fluorescent intensity; MFI units) on EV samples generated from the four hBM-MSC donors collected at the three collection time points (days 1, 13, and 25), albeit statistical differences during this production timeline were detected (Fig. 6b). CD9 antigen levels were statistically different between day 1 (2184 ± 172 MFI, mean ± S.E.M.) as compared to day 13 (1440 ± 250 MFI, mean ± S.E.M.) (*p* < 0.001) and day 25 (1400 ± 456 MFI, mean ± S.E.M.) (*p* < 0.0001), whereas no differences were observed between day 13 and day 25 (*p* < 0.1). A significant increase in CD81 antigen expression level was observed between day 1 (2793 ± 292 MFI, mean ± S.E.M.) and day 13 (3469 ± 330 MFI, mean ± S.E.M.) (*p* < 0.001) as well as compared to day 25 (3373 ± 419 MFI, mean ± S.E.M.) (*p* < 0.01), while no statistical difference was observed between day 13 and day 25 (*p* = 0.9). A significant increase in the intensity level of CD63 antigen level was also observed at day 25 (5329 ± 619 MFI, mean ± S.E.M.) compared to day 1 (2584 ± 198 MFI, mean ± S.E.M.) and day 13 (2397 ± 429 MFI, mean ± S.E.M.) (*p* < 0.0001). Regardless of these statistical differences between EV collection time points, these results show that the expression levels of tetraspanins CD9, CD63, and CD81 are highly detectable at every time point and from every hBM-MSC donor.

MSC-known epitopes were also detectable on EV samples by the antibody-bead conjugates including CD44, CD146, CD29, MCSP, CD49E, and CD105 (Fig. 6), mirroring antigens present on the EV producer cells (hBM-MSCs). Among these MSC-reported antigens, CD105, CD29, and CD49E showed a significant decrease in expression level between day 1 to day 13 as well as between day 1 and day 25, but remained unchanged between day 13 and day 25. For example, CD105 antigen levels were measured at 962 ± 51 MFI (mean ± S.E.M.) at day 1 as compared to day 13 and day 25 where intensity levels reached 334.99 ± 100.71 MFI (mean ± S.E.M.) and 216 ± 87 MFI (mean ± S.E.M.), respectively, highlighting a statistical difference between day 1 versus day 13 (*p* < 0.01) and day 25 (*p* < 0.01). In contrast, CD44 antigen level increased significantly between day 1 (1900 ± 280 MFI, mean ± S.E.M.) and day 25 (2357 ± 306 MFI, mean ± S.E.M.) (*p* < 0.05) as well as between day 13 (1834 ± 366 MFI, mean ± S.E.M.) and day 25 (2357 ± 306 MFI, mean ± S.E.M.) (*p* < 0.05).

MHC class I (HLA-ABC) and class II (HLA-DR) antigens as well as immune co-stimulatory molecule CD40 were among the 37-specific epitopes analyzed by the flow-based assay. HLA-DR antigen was detected at very low and variable APC fluorescence intensity level among all EV samples, ranging from 0 to 102 MFI (Supplementary Table 4). When averaged, the four hBM-MSC donors presented a MFI of 14 ± 3 (mean ± S.E.M.) at day 1, 19 ± 10 (mean ± S.E.M.) at day 13, and 32 ± 23 (mean ± S.E.M.) at day 25, where no statistical differences were observed between the time points (Fig. 6b). Similarly, a low and variable APC fluorescence intensity level was detected among all EV samples for HLA-ABC, ranging from 30 to 356 MFI (Supplementary Table 4). When averaged, the four hBM-MSC donors presented a MFI of 210 ± 55 (mean ± S.E.M.) at day 1, 61 ± 13 (mean ± S.E.M.) at day 13, and 70 ± 16 (mean ± S.E.M.) at day 25, where no statistical differences were observed between the time points (Fig. 6b). CD40 antigen was detected consistently on EV samples from all four donors at all three time points, although a wide range of level was detected (108–1181 MFI) (Supplementary Table 4). When averaged, the four hBM-MSC donors presented a MFI of 783 ± 175 (mean ± S.E.M.) at day 1, 182 ± 28 (mean ± S.E.M.) at day 13, and 348 ± 103 (mean ± S.E.M.) at day 25, where statistical differences were found between day 1 and day 13 (*p* < 0.01) as well as between day 1 and day 25 (*p* < 0.05). Finally, detectable levels of tyrosine-protein kinase transmembrane receptor ROR1 was observed from all EV samples analyzed, ranging from 129 ± 23 MFI (mean ± S.E.M.) at day 1 to 135 ± 42 MFI at day 13 and 472 ± 221 MFI at day 25, where no significant differences between time points were observed. Other antigens were detected at a very low APC fluorescence intensity levels (all at < 200 MFI) and comprised CD56, SSEA-4, CD41B, CD24, CD142, and CD20 epitopes; suggesting either a lack of expression (very close to background) or a very low expression linked to a particular hBM-MSC donor or time point of EV collection.

### EVs produced by hBM-MSCs inoculated in the hollow-fiber cell bioreactor system yielded nanovesicles with an immuno-modulatory payload

**Fig. 7:**
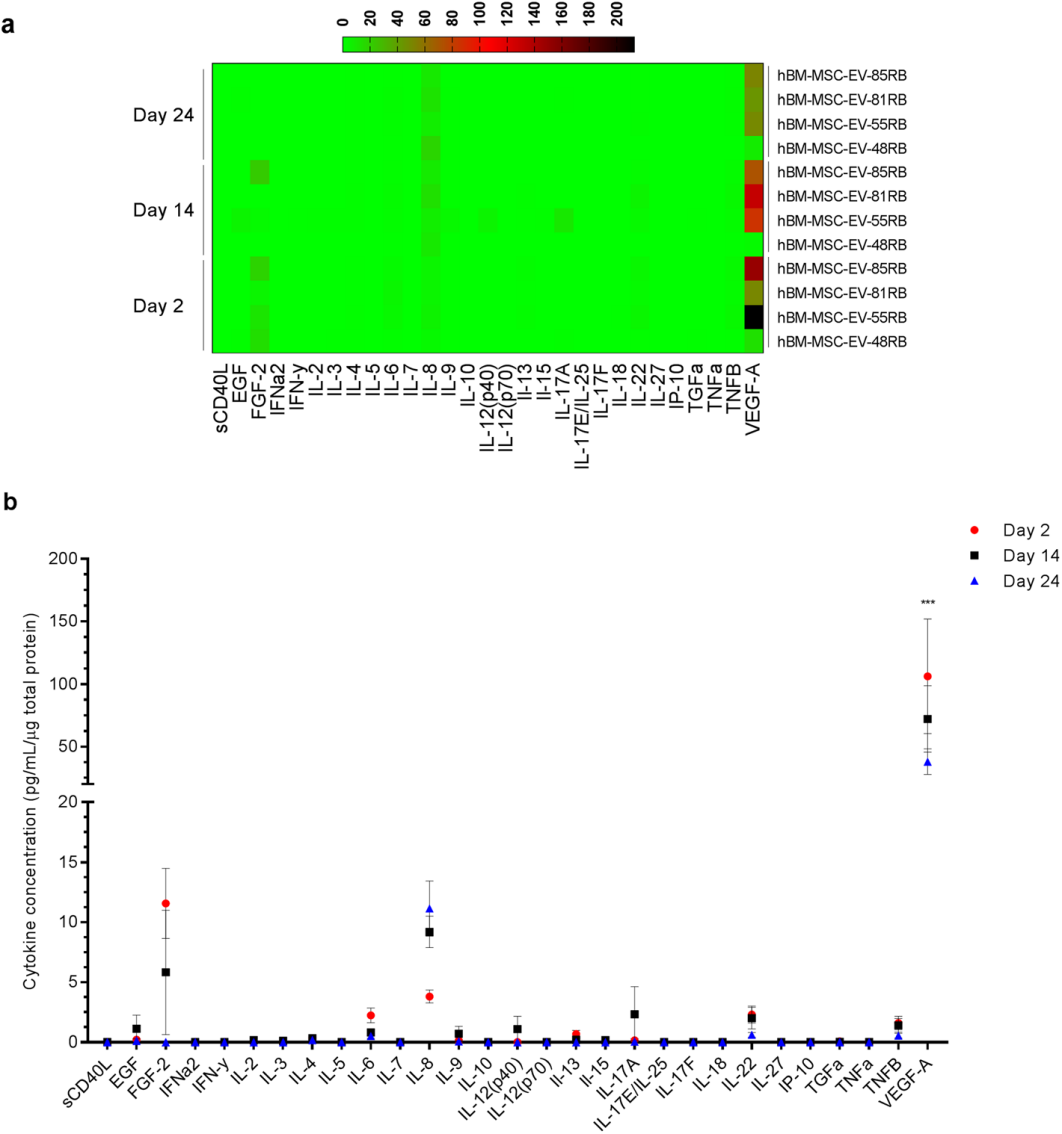
Analysis of EVs produced in the hollow-fiber system from hBM-MSCs confirms an immune-modulatory cargo profile. **a** Milliplex cytokine analysis of EVs isolated from each hBM-MSC donors (*N* = 4 donors; hBM-MSC-81RB/48RB/55RB/85RB) over several days (days 2, 14, and 24) confirms packaging of anti-inflammatory and angiogenic factors. Protein lysates were generated from EV samples collected at days 2, 14, and 24 of the 25-day EV production period. Heat map showing the analysis of 29 cytokines and growth factors from the four donors at the three collection time points. **b** Quantitative analysis of the cytokine and growth factor detection of the mean of the four hBM-MSC donors (*N* = 4 donors; hBM-MSC-81RB/48RB/55RB/85RB) at the three time points (days 2, 14, and 24) is represented. Each dot represents *N* = 4 donors, each analyzed in duplicates. Statistical significance was analyzed by a two-way analysis of variance (ANOVA) with Tukey’s post hoc analysis. A *p* value of < 0.05 was considered statistically significant and significant differences are marked with a triple asterisk (*p* < 0.001)

Next, the cargo content of EVs was investigated for a panel of 29 cytokines/chemokine and growth factors known as biomarkers of inflammation and immune responses. Protein lysates from EVs from the four hBM-MSC donors collected at the three collection time points (days 2, 14, and 24) were individually analyzed by immunology multiplex assay (Fig. 7a). Among the 29 molecules of interest analyzed, IL-6, IL-8, and VEGF-A factors were detected consistently from all EV samples, irrespective of the donor ID and the time point of collection (Fig. 7 and Supplementary Table 5). Notably, detection of pro-inflammatory cytokine IL-6 decreased over time (2.2 ± 0.6 pg/mL/μg protein at day 2 vs. 0.8 ± 0.3 pg/mL/μg protein at day 14 vs. 0.5 ± 0.2 pg/mL/μg protein at day 24), albeit very low levels were detected in additional to no statistical differences observed (Fig. 7b). Interestingly, higher levels of immuno-regulatory cytokine IL-8 were detected and increased over time (3.8 ± 0.5 pg/mL/μg protein at day 2 vs. 9.2 ± 1.3 pg/mL/μg protein at day 14 vs. 11.2 ± 2.3 pg/mL/μg protein at day 24), although the trend was not statistically significant (Fig. 7b).

Importantly, the level of angiogenic and immune-modulatory protein VEGF-A was highly detectable at day 2 (106.2 ± 45.8 pg/mL/μg protein) as compared to day 14 (72.2 ± 26.6 pg/mL/μg protein) and day 24 (38.0 ± 10.2 pg/mL/μg protein), with a statistical downward trend observed from day 2 to day 24 (*p* < 0.0001) (Fig. 7b). Regardless of these statistical differences between days of collection, these results show that VEGF-A is highly detectable at every time point of EV collection. Other molecules among the panel of 29 cytokines/chemokines and growth factors were either non-detectable or detectable at low levels in addition to presenting varying levels among the hBM-MSC donors and the EV collected at the different time points.

## Discussion

Compelling evidence shows that EVs are likely responsible for mediating the therapeutic effect of human mesenchymal stromal cells in a variety of clinical contexts. Therefore, progress with translation of EV therapeutics to clinical settings is required and relies on the manufacturing of clinically relevant EV doses at high-quality requirements. To our knowledge, our work is the first comprehensive study providing extensive characterization of EVs derived from human bone marrow-derived MSCs produced in the hollow-fiber cell bioreactor system under a GMP-compliant environment using serum- and xeno-free certified culture medium for EV production. With increased surface area for cell seeding, the hollow-fiber cell bioreactor system allows a high cell seeding density under a homogeneous controlled-culture environment (temperature and oxygen levels), whereby cell-cell interactions are promoted. The hollow-fiber cell bioreactor from FiberCell Systems operates in a perfusion mode and thus no depletion of glucose, nor its by-product (lactate), are left unmanaged. Surrogates of cell homeostasis (pH, glucose, and lactate measurements) monitored throughout the MSC incubation period into the bioreactor confirmed the steady-state conditions of the MSCs. Additionally, bioreactor-harvested MSCs showed high viability, as well as typical MSC characteristics, including trilineage mesoderm differentiation capacity (osteocytes, adipocytes, and chondrocytes) and MSC-like immunophenotyping profile. These data are in accordance with reports using hollow-fiber bioreactor systems for the expansion of clinical grade manufacturing of human MSCs where it was shown that stem cell properties were maintained throughout the culturing process [30–34]. Noteworthy, 3D environments are also favorable cell culture systems compared to 2D polystyrene surfaces, as they are known to promote the translational value of therapeutics and increase cell functionality to better mimic their native state in vivo [16, 35–37].

In our work, the lab-scale hollow-fiber cell bioreactor from FiberCell Systems was used for the continuous production of EVs over 25 days from primary human BM-MSCs where the culture pipeline was geared towards the production of an EV-rich cell-conditioned medium. This was achieved by incubating the human MSCs with the RoosterCollect-EV medium from RoosterBio during the entire 25-day EV production timeline. The RoosterCollect-EV medium is engineered for EV collection (i.e., low particle content, xeno-free, protein free, chemically defined) rather than for cell growth. With this EV production platform, our 20-mL sampling scheme over the 25 days of EV production led to a collection of 500 mL of serum- and xeno-free EV-rich medium per MSC donor. As quality was maintained throughout the 25-day EV manufacturing process, the daily harvest of 20 mL of EV-rich medium from the hollow-fiber system could therefore be pooled and purified to generate a bulk EV sample, as demonstrated in this study. Given that the lowest number of particles/mL obtained from a 20-mL aliquot of EV-rich medium was 2.18 × 10^9^ particles/mL based on all the samples analyzed, this would translate to a total of 5.5 × 10^10^ particles/mL, therefore exceeding clinical requirements. Similarly, Watson et al. used the hollow-fiber cell system from FiberCell Systems to successfully produce EVs from HEK293 (a human embryonic kidney cell line) expressing a fusion protein (heterodimeric IL-15 and lactadherin complexes) [14]. In contrast to our work, the published methodology by Watson et al. was based only on a 48-h EV production scheme from the engineered HEK293 cells.

Human MSCs are the most popular non-hematopoietic stem cell type for disease prevention and treatment tested in clinical trials. They display low immunogenicity, as indicated by the expression of a very low level of human leukocyte antigens (HLA), and fail to stimulate the allogeneic immune responses [22, 38, 39]. In our study, immunophenotyping analysis of human MSCs was conducted pre- and post-bioreactor seeding to assess whether the MSC surface marker profile would be altered by the bioreactor system conditions. Immunophenotyping analysis by flow cytometry of the four MSC donors post-bioreactor processing showed that the cells retained their MSC-typical phenotypic characteristics, including positive expression of CD73 and CD90 markers and negative expression of HLA-DR, CD11b, CD19, and CD45 antigens, highlighting unchanged expression profile for these markers under the bioreactor incubation conditions. Interestingly, profiling differences were observed for the CD34 and CD105 antigens post-bioreactor processing. The latter antigen showed no expression level and the former showed a slight increased in expression, which was consistent across all four MSC donors. Supporting our results, a compelling body of evidence suggests that CD105 expression can be altered by cell culture conditions, including serum-free medium cultivation as well as 3D environments [12, 40, 41]. For example, Mark and collaborators observed a marked 50% reduction of CD105 expression level when MSCs were cultured under serum-free conditions as compared to serum-containing conditions [41]. To account for this difference, the authors suggested that the CD105 expression variation could be influenced by the composition of the growth medium; a conclusion also supported by Bakopoulou and collaborators where a decreased in CD105 expression level was reported for oral MSCs grown under serum-free medium condition [40]. In accordance, a study by Brohlin et al. showed that bone marrow- and adipose-derived MSCs at late passage showed a significant reduction in expression of CD105 in serum-free medium as compared to serum-containing medium, whereas no change was observed for CD73 and CD90 antigens [42]. Noteworthy, CD105-negative human MSCs have been reported to exhibit a stronger immunomodulation capacity than their CD105-positive counterparts, thereby confirming that CD105-negative MSCs are still functional [43].

When post-bioreactor MSCs were seeded back into their normal culture medium in a 2D adherent state (flask-based), cells were shown to completely restore their CD105 levels up to a degree matching the expression level of the pre-bioreactor processed cells. Akin to our results for the plasticity of the CD105 expression level, Qu et al. observed similar results where MSCs re-expressed CD105 after the cells grown under serum-free conditions were seeded back into serum-containing medium [44]. Noteworthy, in contrast to the cells, analysis of the EV immunophenotyping surface marker profile did show the presence of CD105 expression level on EVs produced throughout the 25-day production scheme, suggesting that the MSCs still produced CD105 when inoculated into the bioreactor, but regulated its expression towards the EVs instead of the cellular membrane. Further investigation is required to understand the mechanistic effect underlying having detectable levels of CD105 onto the EVs, but not on the membrane of the producer cells.

Alteration to the CD34 expression level following hBM-MSC incubation into the bioreactor was also observed in our study where levels of this antigen were found to be consistently increased for the four MSC donors post-bioreactor processing. Even though CD34 is a well-accepted hematopoietic identity marker, accumulated evidence support CD34 being expressed on a multitude of non-hematopoietic cell types, including MSCs and vascular endothelial progenitors [45–47]. Therefore, the notion that CD34 serves as a negative marker for MSC characterization, as initially described in the minimal set of standard criteria for MSCs by the International Society of Gene and Cell Therapy [26], is currently being challenged by literature showing CD34 being expressed in tissue-resident MSCs and under specific cell culture conditions [48]. Indeed, cell culture-associated loss or gain of CD34 expression level has been shown in multiple reports, including evidence from Bakopoulou’s study where CD34 expression on alveolar MSCs was reported to increase gradually during prolonged culture expansion [40]. Interestingly, from the same group, the CD105 expression level on alveolar MSCs was substantially downregulated with passaging which was more pronounced in the serum-free condition as compared to the serum-containing cultures [40]. In line with CD34 and CD105 expression levels being influenced by cell culture states, Bellagamba et al. showed that culturing MSCs as spheroids altered the expression of both markers where CD34 was increased and CD105 was lacking [35]. Importantly, the hollow-fiber cell bioreactor is a system by which cells can be cultured at tissue-like densities over long periods, thus favoring spheroid formation and functionality such as immuno-modulatory and angiogenic properties [49]. As CD34 expression has been associated with particular MSC characteristics such as higher colony-forming efficiency and long-term proliferative capacity [50–52], further investigations into the MSC CD34+ subset population detected after bioreactor processing would be required to understand whether these cells possess distinct characteristics.

Time-based characterization of EVs revealed generally consistent physical (EV size and concentration) and chemical (EV immuno- and glyco-profile) properties between donors, with an observable trend between the early time point of collection (days 1–2) vs the interim (days 13–14) and end of production (days 24–25) for molecular and glycan components. Specifically, differences in expression of molecular markers (CD105, CD49E, CD9, CD40, CD81, CD29, and VEGF-A) and glycan-binding lectins (ConA, LCA, AAL, ECL, PNA, and RCA) were not significantly different at days 13 and 25, while significantly different from day 1, suggesting a potential desired window of EV production between days 13 and 25 to ensure product consistency. All three tetraspanins (CD9, CD63, and CD81) were consistently detected during EV production, confirming EV identity produced by hBM-MSCs under the bioreactor conditions. Absence or very low presence of immunogenicity markers, such as HLA-DR and HLA-ABC antigens, on EVs reinforced the notion that EVs produced by MSCs in our bioreactor mirror the low immunogenicity profile of the producing cells, a favorable characteristic for clinical use in immune-related applications. Furthermore, CD40 antigen was detected on EVs, further supporting the immuno-modulatory role of MSC-EVs as the CD40 ligand/CD40 pathway is widely recognized for its prominent role in immune regulation and homeostasis. Our previous work identified MSC-known antigens (CD73, CD90, CD44, CD146, and MSCP) on EVs, as well as integrins (CD29e and CD49), which were also confirmed herein [6] with a different cohort of four MSC donors grown under different culture conditions. These results not only highlight consistency for the expression of these markers among multiple MSC donors and culture conditions, but also further confirm that EV profile mirrors the one of the cell origin, which further reinforces the EV-to-cell mirroring notion previously described [6, 53, 54].

In this study, we showed that it is not only the cell surface receptor profile of the EV producer cell that is reflected onto the EV profile, but also its molecular cargo content. EVs produced from MSCs can affect immunological and inflammatory pathways by signaling to responding cells via the release of their cargo content. This paracrine-mediated effect through the release of immune factors comprising the EV cargo content was underlined in this work where low levels of pro-inflammatory cytokines IL-6 and TNF-beta were detected, whereas higher levels of angiogenic/immuno-modulatory factors VEGF-A and IL-8 were measured. Similar findings by Kordelas and collaborators were reported where EVs used for treatment were selected based on their molecular content (i.e., higher anti-/pro-inflammatory cytokine content) [21]. Taken together, MSC-EVs display great potential in treating many anti-inflammatory and immuno-modulatory diseases without eliciting unwanted immunogenic reactions. Further analysis of immune-modulatory factors, receptor and cytokines, is warranted to fully understand the immunological and inflammatory effects that MSC-EV signaling has on responding cells, including immune cells.

In conclusion, we have demonstrated that this EV production pipeline based on the hollow-fiber cell bioreactor system can be used for efficient and reproducible EV production from multiple human MSC donors while preserving the stem cell phenotype and functionality of the producer cell. Characterization of the EV producer cell as well as the manufactured EV product is of utmost importance as EV’s therapeutic activity can be influenced by EV molecular constituents reflective of the producer cell status. Utilizing the herein described human MSC-EV production methodology and results, EVs of low immunogenicity and anti-inflammatory characteristics can be generated for future therapeutic purposes.

## Supplementary Information

**Additional file 1: Table S1.** In-process data for working cell banks of hBM-MSCs used in this study.

**Additional file 2: Table S2.** Nanoparticle and protein concentration data analyzed for extracellular vesicles processed for lectin microarray analysis.

**Additional file 3: Table S3.** Nanoparticle tracking analysis summary data of EVs derived from four hBM-MSC donors at the three collection time points.

**Additional file 4: Table S4.** 37-bead multiplex surface epitope analysis of EVs from hBM-MSCs shows maintenance of MSC and EV specific markers over 25 days of EV production as well as presence of immune-related antigens.

**Additional file 5: Table S5.** Analysis of EV samples using the MILLIPLEX MAP human cytokine/chemokine magnetic bead assay.

**Additional file 6: Figure S1.** Immunophenotypic analysis of bioreactor-harvested hBM-MSCs confirms a low positive expression of CD34 antigen, but not HLA-DR. **a)** Flow cytometric analysis of CD34 and HLA-DR antigens from bioreactor-harvested hBM-MSCs from donors hBM-MSC-48RB/81RB/55RB/85RB. Red indicates the cell population stained with the respective antibodies. Blue indicates the cells stained with an IgG isotype control. **b)** Quantification of the percentage of positive cells analyzed by flow cytometry. **Figure S2.** EV production from hBM-MSCs in the hollow-fiber cell bioreactor system yields nanovesicles of small EV size distribution profile. **a)** The mode (i), mean (ii) and the concentration (iii) of EVs are represented for the four hBM-MSC donors (*N* = 4 donors; hBM-MSC-48RB/81RB/55RB/85RB donors) at 3 different time points (days 1, 3 and 25). Each dot represents 5 technical replicates from each the four hBM-MSC donors (hBM-MSC-48RB/81RB/55RB/85RB donors). **Figure S3.** **a**) FPLC injection of 50mL of TFF diafiltrated EV-rich cell conditioned medium (CCM) pooled from 5mL aliquots of CCM harvested each day from the hollow-fiber system from days 1-25 (5mL X 25 days of production) was performed using HiScreen CaptoCore 700 column for EV purification followed by Cleaning In Place (CIP) elution of CCM contamination. The CCM used in this analysis was obtained from donor #hBM-MSC-81RB. EV collection occurred once the UV baseline began to rise indicated by the fraction markers in red. Fractionation was stopped and switched to waste once the UV peak began to fall. CIP was conducted after fractionation to determine the amount of contaminates removed as indicated by the single peak. These data represent n=1 experiment using n=1 donor sample (#hBM‑MSC-81RB). **b**) Nanoparticle tracking analysis (NTA) of the pooled FPLC fractions containing EVs purified by FLPC. Each dot represents 5 technical replicates of donor sample #hBM-MSC-81RB. **c**) Flow cytometric validation of the pooled FPLC fractions containing EVs show CD63-bead purified EVs followed by detection with anti-CD63/CD81/CD9‑APC antibody cocktail, as indicated in blue. The unstained CD63‑bead purified EVs control (no APC antibody cocktail detection) is shown in red and was used to set the negative population. These data represent n=1 experiment using n=1 donor sample (#hBM‑MSC-81RB). **Figure S4.** Transmission electron microscopy (TEM) analysis confirmed the presence of small EVs. EVs from hBM-MSC donor 55RB were assessed by TEM which showed expected morphology and size of small EVs (< 200 nm) (scale bars = 100 or 200 nm as indicated).

## Data Availability

The flow cytometric, milliplex, and nanoparticle tracking analysis datasets are available in the Supplementary section.

## Abbreviations

BM: Bone marrow
CCM: Cell-conditioned medium
ED: Exosome-depleted
EV: Extracellular vesicle
hMSC: Human mesenchymal stromal/stem cells
hBM-MSC: Human bone marrow-derived mesenchymal stromal/stem cells
HLA: Human leukocyte antigen
ISCT: International Society for Cellular Therapy
ISEV: International Society for Extracellular Vesicle
MFI: Median fluorescence intensity
MHC: Major histocompatibility complex
MSC: Mesenchymal stromal/stem cell
NTA: Nanoparticle tracking analysis
PEG: Polyethylene glycol
PFA: Paraformaldehyde
RIPA: Radioimmunoprecipitation assay
SEC: Size exclusion chromatography
