# Supplementary material for: Hollow-fiber bioreactor production of extracellular vesicles from human bone marrow mesenchymal stromal cells yields nanovesicles that mirrors the immuno-modulatory antigenic signature of the producer cell
Source: Stem Cell Res Ther. 2021 Feb 12;12:127. doi: 10.1186/s13287-021-02190-3 (PMC7880218; doi:10.1186/s13287-021-02190-3)
Supplement: Supplementary file 6 — Additional file 6: Figure S1. Immunophenotypic analysis of bioreactor-harvested hBM-MSCs confirms a low positive expression of CD34 antigen, but not HLA-DR. a) Flow cytometric analysis of CD34 and HLA-DR antigens from bioreactor-harvested hBM-MSCs from donors hBM-MSC-48RB/81RB/55RB/85RB. Red indicates the cell population stained with the respective antibodies. Blue indicates the cells stained with an IgG isotype control. b) Quantification of the percentage of positive cells analyzed by flow cytometry. Figure S2. EV production from hBM-MSCs in the hollow-fiber cell bioreactor system yields nanovesicles of small EV size distribution profile. a) The mode (i), mean (ii) and the concentration (iii) of EVs are represented for the four hBM-MSC donors (N = 4 donors; hBM-MSC-48RB/81RB/55RB/85RB donors) at 3 different time points (days 1, 3 and 25). Each dot represents 5 technical replicates from each the four hBM-MSC donors (hBM-MSC-48RB/81RB/55RB/85RB donors). Figure S3. a) FPLC injection of 50mL of TFF diafiltrated EV-rich cell conditioned medium (CCM) pooled from 5mL aliquots of CCM harvested each day from the hollow-fiber system from days 1-25 (5mL X 25 days of production) was performed using HiScreen CaptoCore 700 column for EV purification followed by Cleaning In Place (CIP) elution of CCM contamination. The CCM used in this analysis was obtained from donor #hBM-MSC-81RB. EV collection occurred once the UV baseline began to rise indicated by the fraction markers in red. Fractionation was stopped and switched to waste once the UV peak began to fall. CIP was conducted after fractionation to determine the amount of contaminates removed as indicated by the single peak. These data represent n=1 experiment using n=1 donor sample (#hBM‑MSC-81RB). b) Nanoparticle tracking analysis (NTA) of the pooled FPLC fractions containing EVs purified by FLPC. Each dot represents 5 technical replicates of donor sample #hBM-MSC-81RB. c) Flow cytometric validation of the pooled FPLC fraction [file 13287_2021_2190_MOESM6_ESM.zip › SCRT-Lavoie-Supplemental-Text.docx]

**Supplemental Materials and Methods**

**Transmission Electron Microscopy (TEM)**

EV samples from hBM-MSCs were isolated as described in section 2.6 with slight modifications. Briefly, 5mL of EV-rich cell-conditioned medium (CCM) was used for the precipitation of EVs. The EV pellet was suspended in 200μL of filtered DPBS^-/-^. An equal amount of fixation buffer (filtered DPBS^-/-^ + 4% paraformaldehyde) was added to the EV sample. Each carbon-coated electron microscopy grid (Electron Microscopy Sciences; cat#CF300-CU) was glow discharged for 45 seconds. The grid was allowed to incubate on a drop of 25μL of fixed EVs, which was added on parafilm for 10 minutes at room temperature before it was incubated in uranyless for 1 minute at room temperature. Image capturing was performed on a FEI Tecnai G2 Spirit Twin TEM with a Lab6 emitter, operating at 120kV. The images were acquired with an Eagle camera with a 16k resolution.

**EV purification by Tangential Flow Filtration (TFF) coupled with Fast Protein Liquid Chromatography (FPLC)**

For EV processing by TFF, a total of 125mL of the EV-rich cell conditioned medium (CCM) was utilized. This 125mL CCM sample was obtained from pooling 5mL aliquots of EV-rich CCM harvested each day from the hollow-fiber system from days 1-25 (5mL X 25 days of production) generated from donor #hBM-MSC-81RB. The 125mL sample was diluted with 125mL of filtered DPBS-/- prior to analysis. Diluted CCM (250 mL) was added to a pre-equilibrated Minimate TFF system (Pall Corporation) equipped with a 300kDa Capsule (Cat#OA300C12). The TFF system was run at a flow rate of 50mL/min and the retentate tubing clamp pressure was adjusted until a cross flow pressure of 10‑20psi was achieved. Concentration/diafiltration was allowed to continue until a final volume of 40mL was achieved. The retentate was collected in a 60mL syringe and 10mL of filtered DPBS-/- was added to the TFF system to circulate through the cartridge and collect residual EVs, yielding a final sample volume of 50mL.

For EV Purification, a HiScreen CaptoCore 700 column (GE Lifesciences, Cat# 17548115) installed onto an AKTA Start chromatography system (GE Lifesciences, Cat# 29022094) was utilized. The system was flushed with 20% ethanol, filtered ultrapure water and finally equilibrated with filtered DPBS-/- until the UV Absorbance baseline was stable for 5 minutes. The column was loaded with the TFF retentate (50mL) at a flow rate of 1mL/min and fraction collection began once the UV Absorbance began to plateau. Fractions were collected until the UV Absorbance plateau began to drop. Fractions were then pooled (9 x 5mL fractions) and sterile filtered using a 0.2 micron syringe filter (Pall Acrodisc, Cat#4612), yielding a purified EV sample of approximately 45mL.

**FPLC-purified EV sample analysis by Nanoparticle Tracking Analysis (NTA)**

To determine size distribution and concentration of the EVs purified by FPLC, 250µL of the sample was diluted with 750µL of filtered DPBS-/- prior to nanoparticle tracking analysis performed as per section 2.7 entitled “Nanoparticle tracking analysis (NTA) of EVs using the NanoSight NS300”.

**FPLC-purified EV sample analysis by flow cytometry using CD63 detection reagent**

To validate the EV identity of nanoparticles isolated by FPLC, 900µL of FPLC-purified EV sample was incubated overnight with 100µL of the Exosome Human CD63 Detection reagent (Invitrogen, Cat# 10606D) (i.e. anti-CD63 beads) at 1250rpm (4°C) on a ThermoMixer (Eppendorf). The CD63-bead bound purified EVs were then washed using a DynaMag2 magnet, resuspended with filtered D-PBS-/- and incubated with anti-CD63/CD81/CD9-APC antibody cocktail (MACSplex Exosome Detection Reagent, Cat# 130-108-813) for 1 hour at 1250rpm at room temperature on a Thermomixer. The EV-bound beads were then washed using a DynaMag2 magnet and re-suspended in 500µL of filtered DPBS ‑/- for flow cytometric analysis using the LSRII flow cytometer (BD Biosciences). Up to fifty thousand events per sample were collected. Raw data was analyzed using FlowJo V10 (FlowJo LLC, USA).

**Figure Legend:**

**Figure S1. Immunophenotypic analysis of bioreactor-harvested hBM-MSCs confirms a low positive expression of CD34 antigen, but not HLA-DR. a)** Flow cytometric analysis of CD34 and HLA-DR antigens from bioreactor-harvested hBM-MSCs from donors hBM‑MSC‑48RB/81RB/55RB/85RB. Red indicates the cell population stained with the respective antibodies. Blue indicates the cells stained with an IgG isotype control. **b)** Quantification of the percentage of positive cells analyzed by flow cytometry.

**Figure S2.** **EV production from hBM-MSCs in the hollow-fiber cell bioreactor system yields nanovesicles of small EV size distribution profile. a)** The mode (i), mean (ii) and the concentration (iii) of EVs are represented for the four hBM-MSC donors (N=4 donors; hBM‑MSC‑48RB/81RB/55RB/85RB donors) at 3 different time points (days 1, 3 and 25). Each dot represents 5 technical replicates from each the four hBM-MSC donors (hBM‑MSC‑48RB/81RB/55RB/85RB donors).

**Figure S3. Validation of highly purified EVs by TFF diafiltration coupled with FPLC purification**

**a)** FPLC injection of 50mL of TFF diafiltrated EV-rich cell conditioned medium (CCM) pooled from 5mL aliquots of CCM harvested each day from the hollow-fiber system from days 1-25 (5mL X 25 days of production) was performed using HiScreen CaptoCore 700 column for EV purification followed by Cleaning In Place (CIP) elution of CCM contamination. The CCM used in this analysis was obtained from donor #hBM-MSC-81RB. EV collection occurred once the UV baseline began to rise indicated by the fraction markers in red. Fractionation was stopped and switched to waste once the UV peak began to fall. CIP was conducted after fractionation to determine the amount of contaminates removed as indicated by the single peak. These data represent n=1 experiment using n=1 donor sample (#hBM‑MSC-81RB). **b)** Nanoparticle tracking analysis (NTA) of the pooled FPLC fractions containing EVs purified by FLPC. Each dot represents 5 technical replicates of donor sample #hBM-MSC-81RB. **c)** Flow cytometric validation of the pooled FPLC fractions containing EVs show CD63-bead purified EVs followed by detection with anti-CD63/CD81/CD9‑APC antibody cocktail, as indicated in blue. The unstained CD63‑bead purified EVs control (no APC antibody cocktail detection) is shown in red and was used to set the negative population. These data represent n=1 experiment using n=1 donor sample (#hBM‑MSC-81RB).

**Figure S4. Transmission electron microscopy (TEM) analysis confirmed the presence of small EVs.** EVs from hBM-MSC donor 55RB were assessed by TEM which showed expected morphology and size of small EVs (< 200 nm) (scale bars= 100 or 200 nm as indicated).
