# Supplementary figures and images for: Hollow-fiber bioreactor production of extracellular vesicles from human bone marrow mesenchymal stromal cells yields nanovesicles that mirrors the immuno-modulatory antigenic signature of the producer cell
Source: Stem Cell Res Ther. 2021 Feb 12;12:127. doi: 10.1186/s13287-021-02190-3 (PMC7880218; doi:10.1186/s13287-021-02190-3)

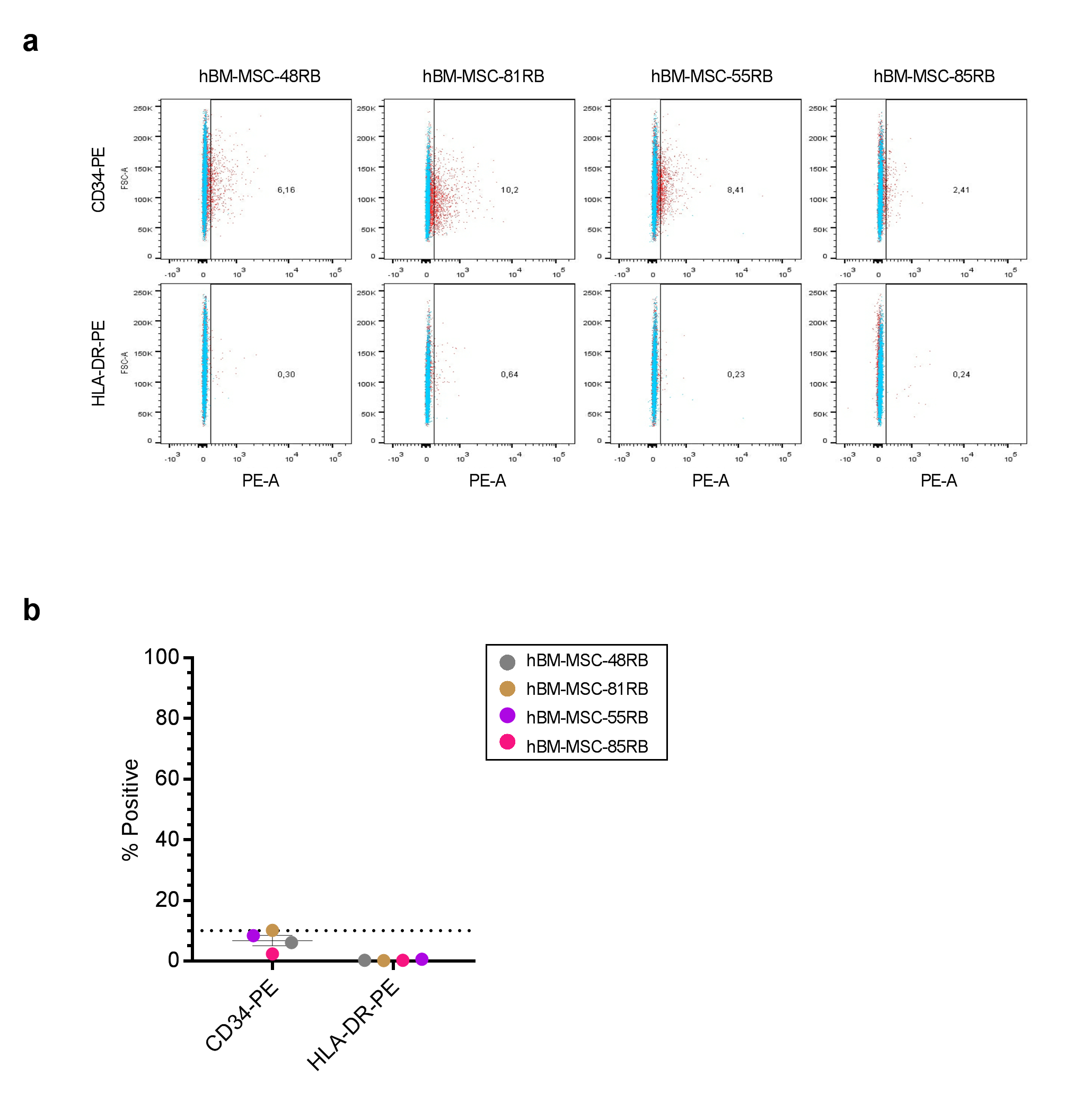

Supplement: Supplementary file 6 — Additional file 6: Figure S1. Immunophenotypic analysis of bioreactor-harvested hBM-MSCs confirms a low positive expression of CD34 antigen, but not HLA-DR. a) Flow cytometric analysis of CD34 and HLA-DR antigens from bioreactor-harvested hBM-MSCs from donors hBM-MSC-48RB/81RB/55RB/85RB. Red indicates the cell population stained with the respective antibodies. Blue indicates the cells stained with an IgG isotype control. b) Quantification of the percentage of positive cells analyzed by flow cytometry. Figure S2. EV production from hBM-MSCs in the hollow-fiber cell bioreactor system yields nanovesicles of small EV size distribution profile. a) The mode (i), mean (ii) and the concentration (iii) of EVs are represented for the four hBM-MSC donors (N = 4 donors; hBM-MSC-48RB/81RB/55RB/85RB donors) at 3 different time points (days 1, 3 and 25). Each dot represents 5 technical replicates from each the four hBM-MSC donors (hBM-MSC-48RB/81RB/55RB/85RB donors). Figure S3. a) FPLC injection of 50mL of TFF diafiltrated EV-rich cell conditioned medium (CCM) pooled from 5mL aliquots of CCM harvested each day from the hollow-fiber system from days 1-25 (5mL X 25 days of production) was performed using HiScreen CaptoCore 700 column for EV purification followed by Cleaning In Place (CIP) elution of CCM contamination. The CCM used in this analysis was obtained from donor #hBM-MSC-81RB. EV collection occurred once the UV baseline began to rise indicated by the fraction markers in red. Fractionation was stopped and switched to waste once the UV peak began to fall. CIP was conducted after fractionation to determine the amount of contaminates removed as indicated by the single peak. These data represent n=1 experiment using n=1 donor sample (#hBM‑MSC-81RB). b) Nanoparticle tracking analysis (NTA) of the pooled FPLC fractions containing EVs purified by FLPC. Each dot represents 5 technical replicates of donor sample #hBM-MSC-81RB. c) Flow cytometric validation of the pooled FPLC fraction [file 13287_2021_2190_MOESM6_ESM.zip › Suppl-Figure1.tif]

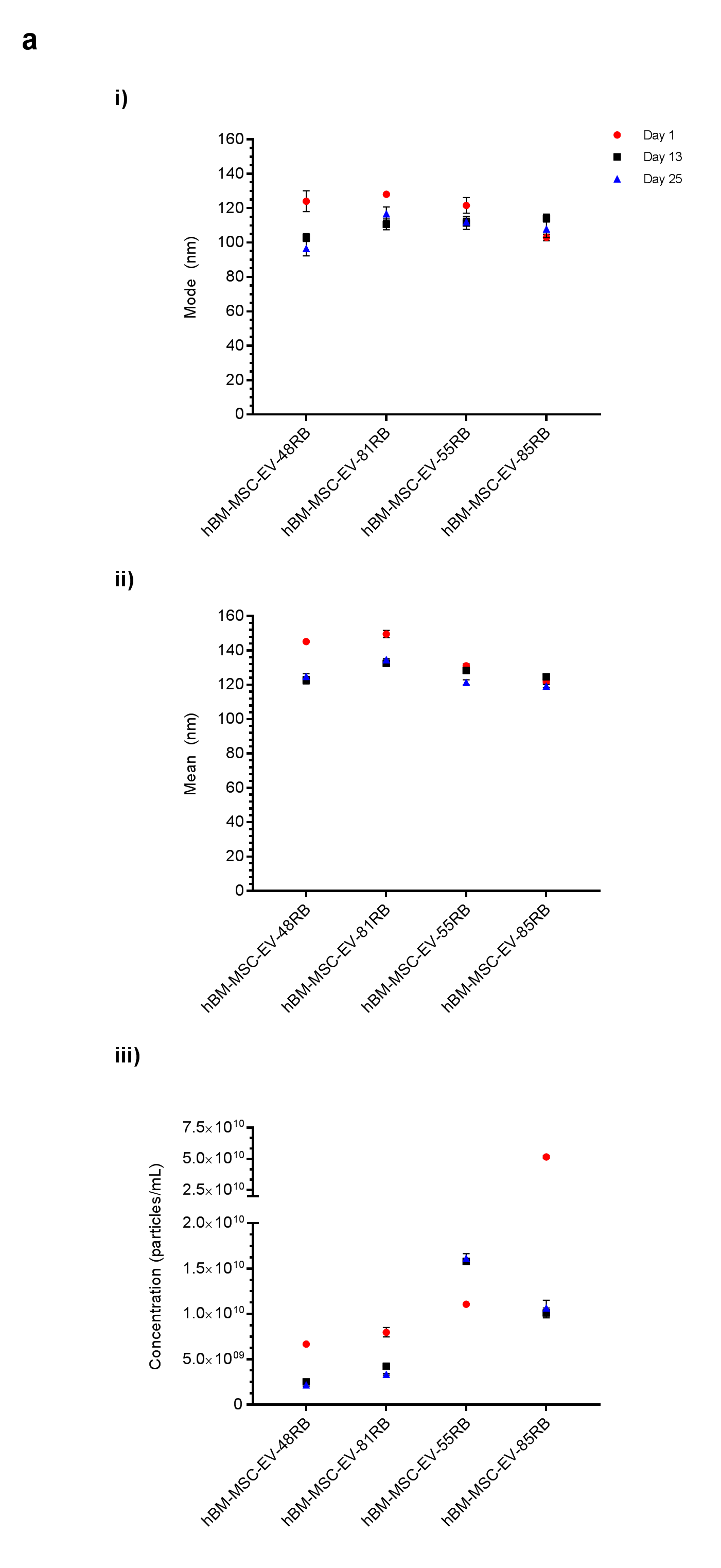

Supplement: Supplementary file 6 — Additional file 6: Figure S1. Immunophenotypic analysis of bioreactor-harvested hBM-MSCs confirms a low positive expression of CD34 antigen, but not HLA-DR. a) Flow cytometric analysis of CD34 and HLA-DR antigens from bioreactor-harvested hBM-MSCs from donors hBM-MSC-48RB/81RB/55RB/85RB. Red indicates the cell population stained with the respective antibodies. Blue indicates the cells stained with an IgG isotype control. b) Quantification of the percentage of positive cells analyzed by flow cytometry. Figure S2. EV production from hBM-MSCs in the hollow-fiber cell bioreactor system yields nanovesicles of small EV size distribution profile. a) The mode (i), mean (ii) and the concentration (iii) of EVs are represented for the four hBM-MSC donors (N = 4 donors; hBM-MSC-48RB/81RB/55RB/85RB donors) at 3 different time points (days 1, 3 and 25). Each dot represents 5 technical replicates from each the four hBM-MSC donors (hBM-MSC-48RB/81RB/55RB/85RB donors). Figure S3. a) FPLC injection of 50mL of TFF diafiltrated EV-rich cell conditioned medium (CCM) pooled from 5mL aliquots of CCM harvested each day from the hollow-fiber system from days 1-25 (5mL X 25 days of production) was performed using HiScreen CaptoCore 700 column for EV purification followed by Cleaning In Place (CIP) elution of CCM contamination. The CCM used in this analysis was obtained from donor #hBM-MSC-81RB. EV collection occurred once the UV baseline began to rise indicated by the fraction markers in red. Fractionation was stopped and switched to waste once the UV peak began to fall. CIP was conducted after fractionation to determine the amount of contaminates removed as indicated by the single peak. These data represent n=1 experiment using n=1 donor sample (#hBM‑MSC-81RB). b) Nanoparticle tracking analysis (NTA) of the pooled FPLC fractions containing EVs purified by FLPC. Each dot represents 5 technical replicates of donor sample #hBM-MSC-81RB. c) Flow cytometric validation of the pooled FPLC fraction [file 13287_2021_2190_MOESM6_ESM.zip › Suppl-Figure2.tif]

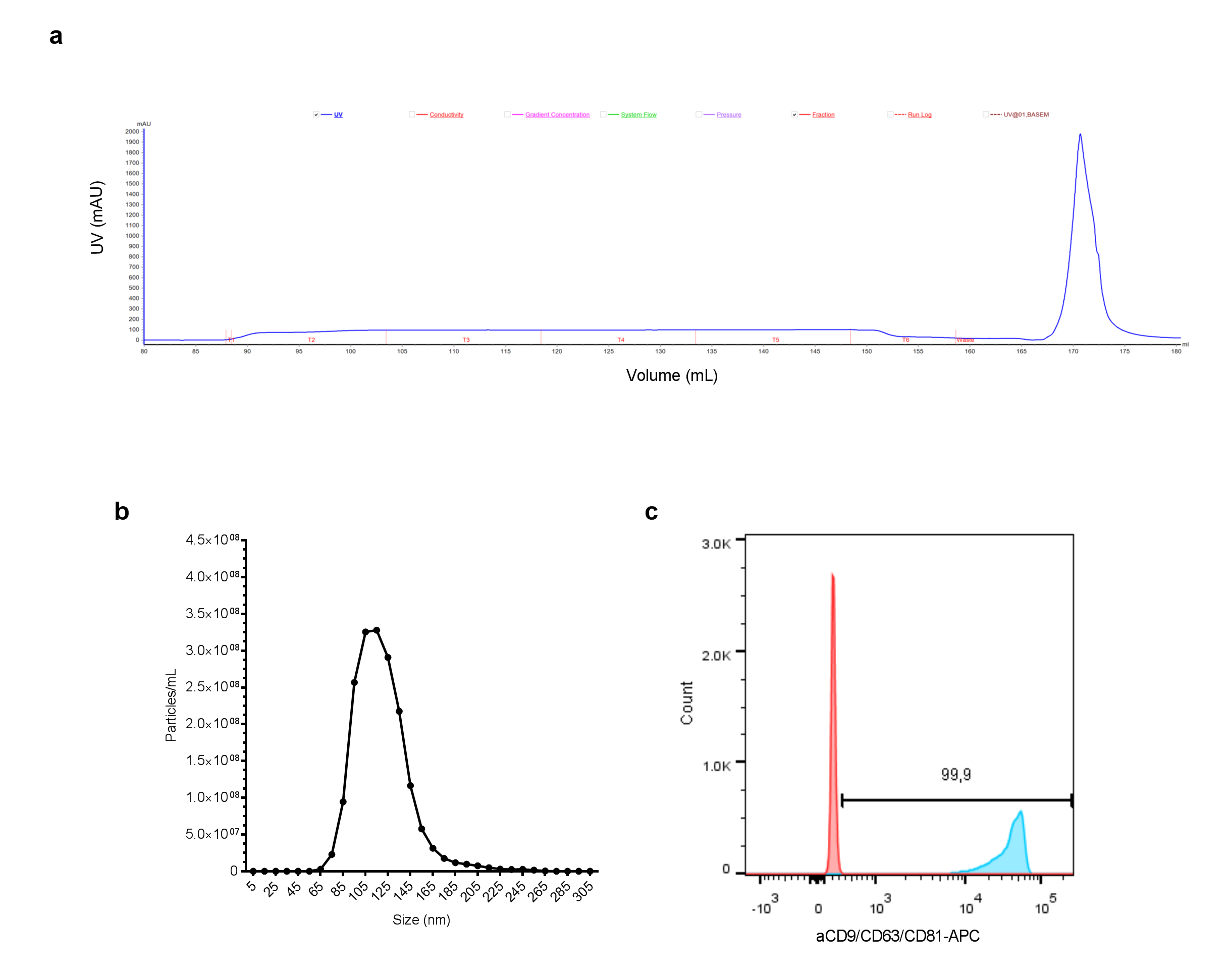

Supplement: Supplementary file 6 — Additional file 6: Figure S1. Immunophenotypic analysis of bioreactor-harvested hBM-MSCs confirms a low positive expression of CD34 antigen, but not HLA-DR. a) Flow cytometric analysis of CD34 and HLA-DR antigens from bioreactor-harvested hBM-MSCs from donors hBM-MSC-48RB/81RB/55RB/85RB. Red indicates the cell population stained with the respective antibodies. Blue indicates the cells stained with an IgG isotype control. b) Quantification of the percentage of positive cells analyzed by flow cytometry. Figure S2. EV production from hBM-MSCs in the hollow-fiber cell bioreactor system yields nanovesicles of small EV size distribution profile. a) The mode (i), mean (ii) and the concentration (iii) of EVs are represented for the four hBM-MSC donors (N = 4 donors; hBM-MSC-48RB/81RB/55RB/85RB donors) at 3 different time points (days 1, 3 and 25). Each dot represents 5 technical replicates from each the four hBM-MSC donors (hBM-MSC-48RB/81RB/55RB/85RB donors). Figure S3. a) FPLC injection of 50mL of TFF diafiltrated EV-rich cell conditioned medium (CCM) pooled from 5mL aliquots of CCM harvested each day from the hollow-fiber system from days 1-25 (5mL X 25 days of production) was performed using HiScreen CaptoCore 700 column for EV purification followed by Cleaning In Place (CIP) elution of CCM contamination. The CCM used in this analysis was obtained from donor #hBM-MSC-81RB. EV collection occurred once the UV baseline began to rise indicated by the fraction markers in red. Fractionation was stopped and switched to waste once the UV peak began to fall. CIP was conducted after fractionation to determine the amount of contaminates removed as indicated by the single peak. These data represent n=1 experiment using n=1 donor sample (#hBM‑MSC-81RB). b) Nanoparticle tracking analysis (NTA) of the pooled FPLC fractions containing EVs purified by FLPC. Each dot represents 5 technical replicates of donor sample #hBM-MSC-81RB. c) Flow cytometric validation of the pooled FPLC fraction [file 13287_2021_2190_MOESM6_ESM.zip › Suppl-Figure3.tif]

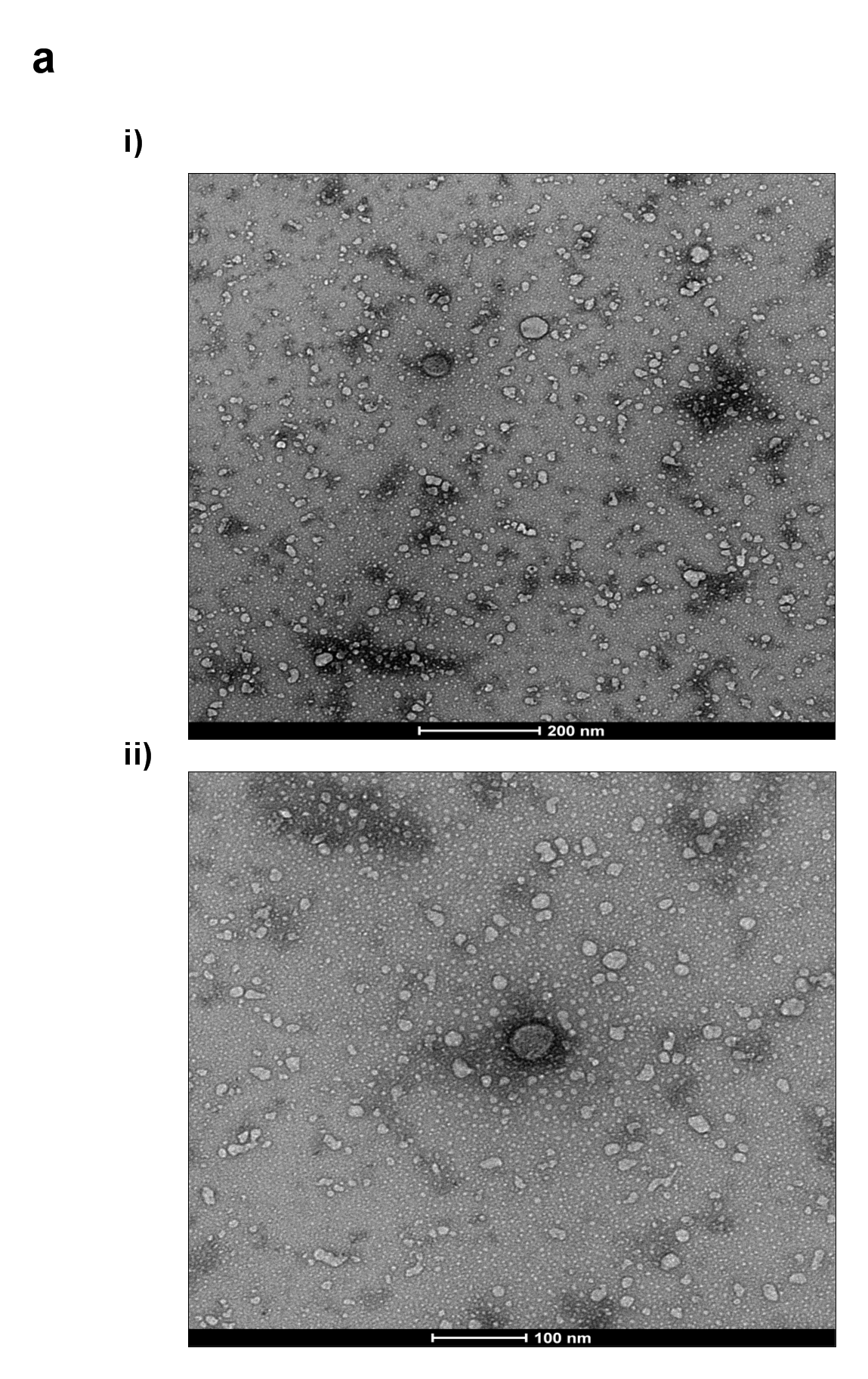

Supplement: Supplementary file 6 — Additional file 6: Figure S1. Immunophenotypic analysis of bioreactor-harvested hBM-MSCs confirms a low positive expression of CD34 antigen, but not HLA-DR. a) Flow cytometric analysis of CD34 and HLA-DR antigens from bioreactor-harvested hBM-MSCs from donors hBM-MSC-48RB/81RB/55RB/85RB. Red indicates the cell population stained with the respective antibodies. Blue indicates the cells stained with an IgG isotype control. b) Quantification of the percentage of positive cells analyzed by flow cytometry. Figure S2. EV production from hBM-MSCs in the hollow-fiber cell bioreactor system yields nanovesicles of small EV size distribution profile. a) The mode (i), mean (ii) and the concentration (iii) of EVs are represented for the four hBM-MSC donors (N = 4 donors; hBM-MSC-48RB/81RB/55RB/85RB donors) at 3 different time points (days 1, 3 and 25). Each dot represents 5 technical replicates from each the four hBM-MSC donors (hBM-MSC-48RB/81RB/55RB/85RB donors). Figure S3. a) FPLC injection of 50mL of TFF diafiltrated EV-rich cell conditioned medium (CCM) pooled from 5mL aliquots of CCM harvested each day from the hollow-fiber system from days 1-25 (5mL X 25 days of production) was performed using HiScreen CaptoCore 700 column for EV purification followed by Cleaning In Place (CIP) elution of CCM contamination. The CCM used in this analysis was obtained from donor #hBM-MSC-81RB. EV collection occurred once the UV baseline began to rise indicated by the fraction markers in red. Fractionation was stopped and switched to waste once the UV peak began to fall. CIP was conducted after fractionation to determine the amount of contaminates removed as indicated by the single peak. These data represent n=1 experiment using n=1 donor sample (#hBM‑MSC-81RB). b) Nanoparticle tracking analysis (NTA) of the pooled FPLC fractions containing EVs purified by FLPC. Each dot represents 5 technical replicates of donor sample #hBM-MSC-81RB. c) Flow cytometric validation of the pooled FPLC fraction [file 13287_2021_2190_MOESM6_ESM.zip › Suppl-Figure4.tif]
